# Expression of IL‐7Rα and KLRG1 defines functionally distinct CD8^+^ T‐cell populations in humans

**DOI:** 10.1002/eji.201847897

**Published:** 2019-03-25

**Authors:** Ester B. M. Remmerswaal, Pleun Hombrink, Benjamin Nota, Hanspeter Pircher, Ineke J. M. ten Berge, René A. W. van Lier, Michiel C. van Aalderen

**Affiliations:** ^1^ Department of Experimental Immunology Amsterdam Infection and Immunity Institute Amsterdam UMC University of Amsterdam Amsterdam The Netherlands; ^2^ Renal Transplant Unit Division of Internal Medicine Academic Medical Centre Amsterdam UMC University of Amsterdam Amsterdam The Netherlands; ^3^ Sanquin Research and Landsteiner laboratory Amsterdam The Netherlands; ^4^ Institute for Immunology University Medical Centre Freiburg Freiburg Germany

**Keywords:** Human T‐cell memory, IL‐7 receptor‐α‐chain, Killer cell lectin‐like receptor subfamily G member 1, Memory precursor effector cells, Short‐lived effector cells, Viral infection

## Abstract

During acute viral infections in mice, IL‐7Rα and KLRG1 together are used to distinguish the short‐lived effector cells (SLEC; IL‐7Rα^lo^KLRG^hi^) from the precursors of persisting memory cells (MPEC; IL‐7Rα^hi^KLRG1^lo^). We here show that these markers can be used to define distinct subsets in the circulation and lymph nodes during the acute phase and in “steady state” in humans. In contrast to the T cells in the circulation, T cells derived from lymph nodes hardly contain any KLRG1‐expressing cells. The four populations defined by IL‐7Rα and KLRG1 differ markedly in transcription factor, granzyme and chemokine receptor expression. When studying renal transplant recipients experiencing a primary hCMV and EBV infection, we also found that after viral control, during latency, Ki‐67‐negative SLEC can be found in the peripheral blood in considerable numbers. Thus, combined analyses of IL‐7Rα and KLRG1 expression on human herpes virus‐specific CD8^+^ T cells can be used to separate functionally distinct subsets in humans. As a noncycling IL‐7Rα^lo^KLRG1^hi^ population is abundant in healthy humans, we conclude that this combination of markers not only defines short‐lived effector cells during the acute response but also stable effector cells that are formed and remain present during latent herpes infections.

## Introduction

Following viral challenge, CD8^+^ T cells with different phenotypes and functions can be found in experimentally infected mice. Acute phase IL‐7 receptor α chain (IL‐7Rα)^lo^ and Killer cell lectin‐like receptor subfamily G member 1 (KLRG1)^hi^ CD8^+^ T cells die‐off after the expansion phase by apoptosis during the contraction phase, and were thus termed short‐lived effector cells (SLECs). In contrast IL‐7Rα^hi^KLRG1^lo^ cells have an increased propensity to persist in the memory phase, and were thus named memory‐precursor effector cells (MPECs) 1, 2, 3. Further, early during the acute phase, recently primed T cells displaying an IL‐7Rα^lo^KLRG1^lo^ early effector cell (EEC) phenotype are found from which both MPECs and SLECs can form 4. This latter observation accords well with complementary data from different approaches demonstrating that effector and memory cells can be generated from a single antigen‐activated precursor 5, 6, 7. The fate of virus‐specific CD8^+^ T cells is determined by transcriptional regulation. Transcription factors, like T‐bet and eomesodermin (Eomes), are crucial regulators of effector and memory formation 8. Despite the fact that T‐bet and Eomes display a high degree of structural homology, as well as functional redundancy, murine T‐bet was found to be more relevant to the generation of effector cells during the acute phase of infection, whereas Eomes is important to the persistence of memory cells and the generation of secondary responses 9, 10.

Importantly, mice and men differ substantially with regard to both lifespan and the range of pathogens with which they have coevolved. These factors possibly also have affected T‐cell memory formation and memory homeostasis, especially when it concerns responses to persistent viruses such as EBV and human CMV (hCMV) that establish lifelong latent infection after the acute phase. Moreover, given the large time span in which such viruses have coevolved with humans and the distinct modes of infection exhibited by each virus, the antiviral T‐cell responses may have adapted in order to provide a defence specifically tailored to best control these distinct infections. Supporting evidence for this notion is, among others, provided by our observation that HOBIT, a BLIMP‐1 homolog regulating effector functions in murine NKT cells 11, is specifically expressed in human cytolytic CD8^+^CD45RA^+^CD27ˉ T cells, a population selectively induced in hCMV‐infected people 12.

Combinations of different cell surface markers have been used to define total and virus‐specific human CD8^+^ T‐cell subsets. Combinations of CD45RA, CCR7, CD28, and CD27 are commonly being employed to define naïve (CD45RA^+^CCR7^+^CD28^+^CD27^+^), central memory (CD45RAˉCCR7^+^CD28^+^CD27^+^), effector memory (CD45RAˉCCR7ˉCD28^+^CD27^+^), and effector‐type cells (CD45RA^+^CCR7ˉCD28ˉCD27ˉ, also referred to as T_EM_RA, for a review see 13). However, since this marker combination cannot be used for the definition of murine T cells subsets, a comparison of subset development between experimentally infected mice and naturally infected humans is cumbersome. For this reason, we here determined IL‐7Rα/KLRG1 phenotypes of human CD8^+^ T cells targeting different persisting viruses in both latently infected healthy individuals and primary hCMV‐ and EBV‐infected kidney allograft recipients. In addition, we analyzed if different IL‐7Rα/KLRG1 expression patterns associate with different functional profiles of cells.

CD8^+^ T cells with all possible combinations of IL‐7Rα/KLRG1 expression were detectable in blood and lymph nodes of healthy adults, of which the vast majority was Ki‐67‐negative (noncycling). Furthermore, IL‐7Rα/KLRG1 expression patterns define different functional CD8^+^ T cells subtypes with unique transcriptomes. IL‐7Rα‐negative cells were generally expressing markers that befit a cytotoxic functional profile. Among these, the relatively small IL‐7Rα^lo^KLRG1^lo^ population uniquely harbors Ki‐67^+^ (cycling) cells in healthy asymptomatic individuals. Further, in all study cohorts, substantial numbers of CD8^+^ T cells were detected with an IL‐7Rα^lo^KLRG1^hi^ “SLEC” phenotype, expressing high amounts of T‐bet and granzyme B, that persist in a Ki‐67‐negative “steady state” long after viral control. Importantly, these cells are enriched in the hCMV‐specific population and are largely excluded from the lymph nodes. Thus, in humans, a stable pool of quiescent CD8^+^ “vigilant” effector‐type cells, phenotypically corresponding to murine short‐lived effector cells, is abundant in the circulating compartment. Such cells were seen significantly less often among human EBV‐specific memory populations, and by monitoring T‐cell responses during primary infection we found that these distinctions per herpes virus‐targeted response are installed early during the acute phase.

## Results

### IL‐7Rα and KLRG1 expression patterns identify cytotoxic and noncytotoxic memory cells

**Figure 1:**
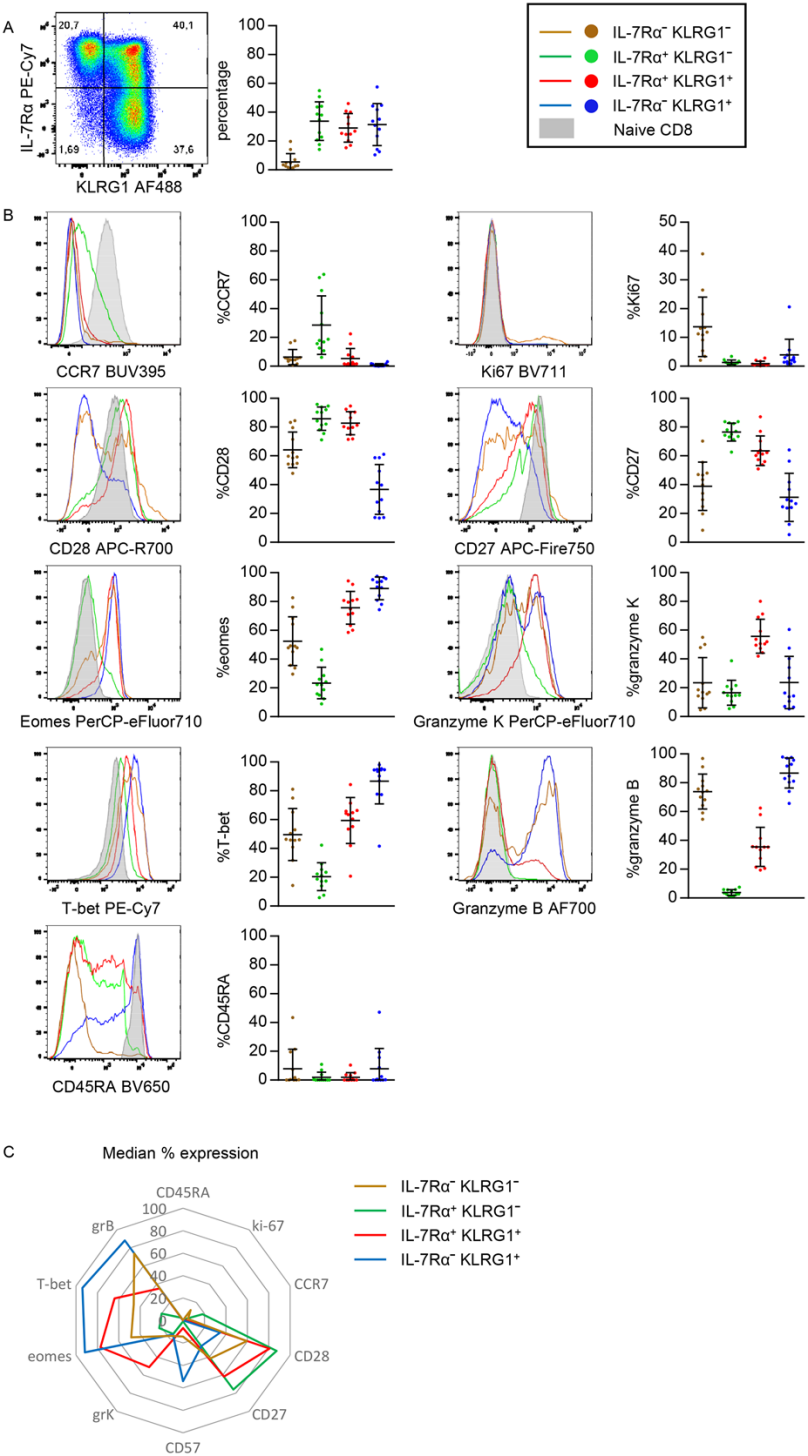
Distribution and phenotype of KLRG1/IL‐7Rα‐defined subsets in non‐naive CD8^+^ T cells. (A) (left) Flow cytometric analysis of KLRG1 and IL‐7Rα expression in nonnaive CD8 T cells and (right) distribution of KLRG1/IL‐7Rα‐defined subsets in nonnaive CD8^+^ T cells from the peripheral blood of 12 healthy donors. (B) Percentages of expression of CCR7, CD28, Eomes, T‐bet, CD45RA (left column top to bottom) and Ki‐67, CD27, granzyme K, granzyme B, and CD57 (right column top to bottom). (C) Spider plot of the mean expression of data shown in B. H‐SNE analysis of the data can be found in Supporting Information Figure 2. Data shown (mean ± SD) are representative of three independent experiments with *n* = 8, 2, and 4 donors per experiment. Twelve unique donors are shown.

We first enumerated the four IL‐7Rα/KLRG1‐defined subsets within the total circulating CD8^+^ T‐cell pool of EBV and hCMV‐seropositive healthy adults (after exclusion of the naïve population). We specifically selected these individuals since both hCMV and EBV significantly impact the makeup of the circulating CD8^+^ T‐cell pool (i.e. smaller naïve and central‐memory populations and large T_EM_RA populations) and because the majority of the adults is latently infected with these viruses (hCMV ∼50–60% and EBV 80–90%) 14, 15, 16. In these individuals that had no clinical and/or laboratory signs of acute viral infection, we detected substantial numbers of KLRG1‐expressing cells. These were either displaying an IL‐7Rα^hi^ double‐positive effector cell (DPEC) phenotype or an IL‐7Rα^lo^ (in mice defined as SLEC) phenotype, in the circulating nonnaïve pool (Fig. 1A).

A considerable proportion of the cells contained within the KLRG1^+^ subsets, especially those with an IL‐7Rα^lo^ phenotype, express effector molecules like T‐bet and granzymes (Fig. 1B and Supporting Information Figs. 2 and 3). Therefore, these cells closely approximate CD28‐negative effector type (T_EM_RA) cells 14. IL‐7Rα^hi^KLRG1^lo^ MPEC cells instead displayed markers that fit a classic noncytotoxic memory phenotype, with a considerable amount expressing the lymphoid tissue‐homing receptor CCR7, the costimulatory receptors CD28 and CD27, while showing a low abundance of T‐bet, CD57 and granzymes (Fig. 1B, C, Supporting Information Figs. S2, S3, and S4) 17, 18, 19, 20. Indeed, these cells therefore closely approximate the central‐memory and early‐differentiated effector‐memory subsets 14.

A fourth IL‐7Rα^lo^KLRG1^lo^ subset (previously coined EEC in mice 21), was also detected in the circulation of these healthy individuals, although at much lower frequencies than the other subsets (Fig. 1A). In accordance with their name, these cells indeed displayed all the traits associated with cytotoxic effector function and were in this respect more similar to the KLRG1^+^ subsets than to the IL‐7Rα^hi^KLRG1^lo^ MPECs (Fig. 1B and Supporting Information Figs. 2, S3, and S4). Nevertheless, these cells also shared some features with MPECs with regard to a high expression of CCR7 and CCR4 (Fig. 1B, C, Supporting Information Figs. 2, 3, and 4). Interestingly, when compared to the other subsets, the IL‐7Rα^lo^KLRG1^lo^ cells uniquely contained a subpopulation of Ki‐67^+^ (actively cycling) cells (Fig. 1B, C, Supporting Information Figs. 2, 3, and 4). Finally, these cells also highly expressed HLA‐DR and CD38 (Supporting Information Figs. 3 and 4), further indicative of an activated state.

### Transcriptomic analysis reveals distinct functional specializations of IL‐7Rα/KLRG1‐defined subsets

**Figure 2:**
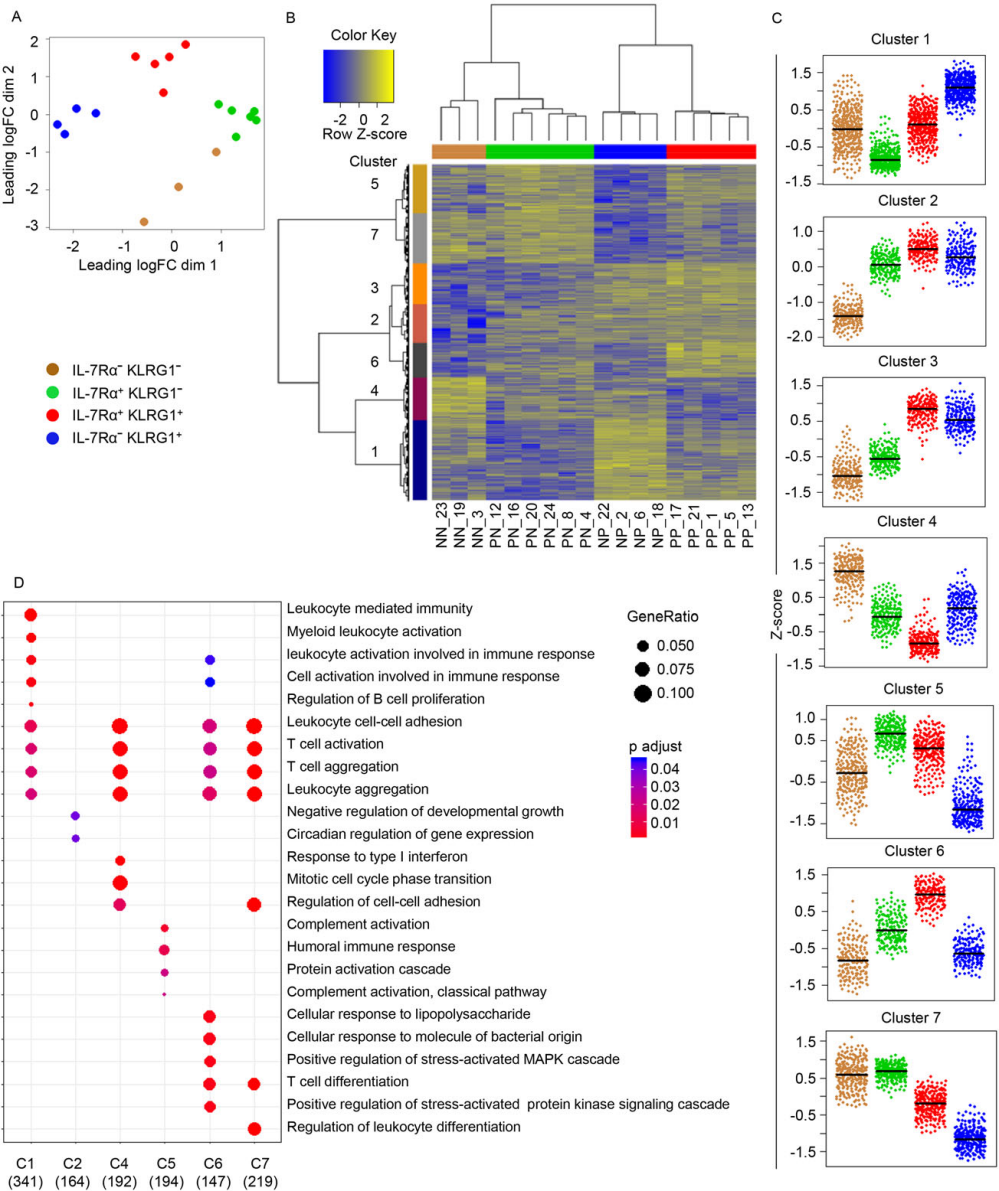
Distinct gene expression profiles by the four T‐cell populations that could be distinguished by the surface expression of IL‐7Rα and KLRG1. Antigen experienced CD8^+^ T cells were FACS‐sorted from buffycoat‐derived PBMCs of *n* = 6 donors according to their IL‐7Rα and KLRG1 expression profiles and subjected to RNA‐seq analysis. (A) Multidimensional scaling of global gene expression data of the four sorted populations (as indicated in key). (B) Hierarchical clustering of analyzed populations and differentially expressed genes. Genes included were significantly differentially expressed between two of the populations. Sample IDs are indicated at the bottom and color‐coded at the top of the graph. On the left side of the plot colors indicate seven gene clusters identified. (C) Gene expression profiles of unique gene clusters by the four heterogenous populations. Displayed scales are Z‐scores of log2 transformed RPKM values. (D) Gene enrichment analysis to link gene clusters with GO terms. Indicated at the bottom are number of genes included per cluster. Data shown (mean ± SD) are from one independent experiments with *n* = 6 donors per experiment. Dot size and color indicates gene ratio and adjusted *p* values (>0.05), respectively.

We determined the transcriptional profile in peripheral blood directly ex vivo of the four populations that could be distinguished by the surface expression of IL‐7Rα and KLRG1. Multidimensional scaling (Fig. 2A) revealed that the expression profiles of all populations segregated according to phenotype rather than by donor. While transcriptional variation among populations sorted based on the expression of at least one marker was minimal, variation among those defined as the double negative population was substantial. Hierarchical clustering (Fig. 2B) demonstrated clustering of samples according to phenotype. Interestingly KLRG1 expression appeared to be the largest denominator as IL‐7Rα^hi^KLRG1^hi^ clustered together with IL‐7Rα^lo^KLRG1^hi^ rather than IL‐7Rα^hi^KLRG1^lo^ populations. Likewise, IL‐7Rα^lo^KLRG1^lo^ clustered with IL‐7Rα^hi^KLRG1^lo^ populations despite their difference in cytotoxic effector traits. Hierarchical clustering revealed seven clusters of differentially expressed genes among the primary T‐cell populations (Fig. 2C). Subsequently, we performed a gene set enrichment aimed at linking gene clusters with GO terms (Fig. 2D). With the exception of cluster 3, all clusters revealed to harbor unique functional characteristics. As such, cluster 1, highly expressed by IL‐7Rα^lo^KLRG1^hi^ cells was enriched for genes mediating leukocyte activation and cluster 4, highly expressed by IL‐7Rα^lo^KLRG1^lo^, was enriched for genes participating in cell cycle. Indeed, befitting the previously observed granzyme B and Ki‐67^+^ (cycling) protein profiles observed in these subsets as described above (Fig. 1).

### The Ki‐67^+^ IL‐7Rα^lo^KLRG1^lo^ effector population concerns activated cells with an effector phenotype

Given the unique presence of actively cycling cells found in immunocompetent asymptomatic individuals in the IL‐7Rα^lo^KLRG1^lo^ population, we were curious to what extent the Ki‐67^+^ EECs differed from the Ki‐67‐negative EECs.

**Figure 3:**
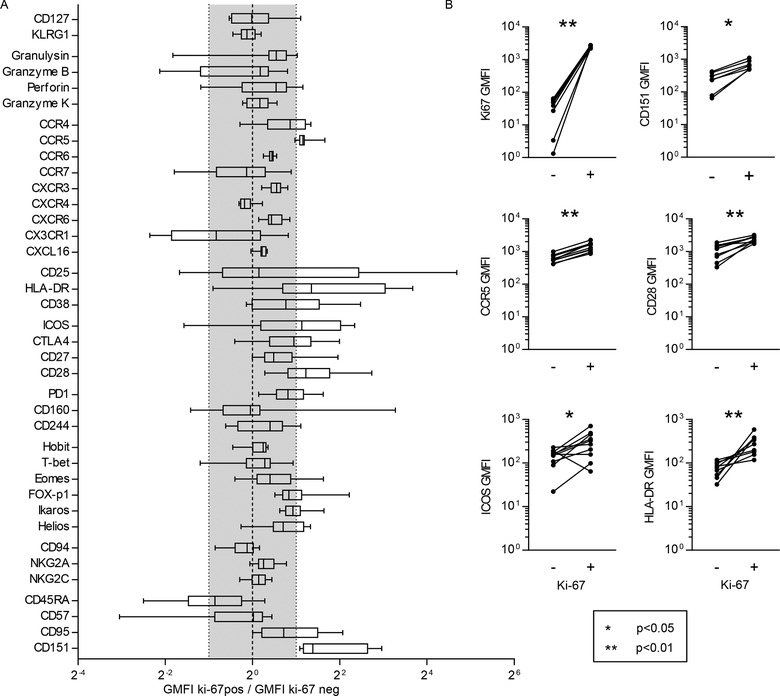
The Ki‐67^+^ IL‐7Rα^lo^KLRG1^lo^ effector population are activated cells with an effector phenotype. (A) Flow cytometric analysis of IL‐7Rα^lo^KLRG1^lo^ nonnaïve CD8^+^ T cells from the PBMCs from ten buffy coats. Box and whiskers min to max plot of the fold change of the geomean fluorescence intensity (GMFI). The gray area represents changes less than twofold. Only when Ki‐67‐expressing IL‐7Rα^lo^KLRG1^lo^ CD8^+^ T cells had a more than twofold higher or lower mean expression (outside the gray area) expression was considered different. (B) MFI of Ki67, CD151, CCR5, CD28, ICOS, and HLA‐DR on ki‐67‐negative (‐) and ki‐67‐expressing (+) IL‐7Rα^lo^KLRG1^lo^ nonnaïve CD8^+^ T cells. Representative histograms and graphs of the individual fold changes of the GMFI of each of the four KLRG1/IL‐7Rα‐defined subsets in nonnaïve CD8^+^ T cells of ten buffy coats compared to the naïve (CD27^+^CD45RA^+^) CD8^+^ T cells of the same sample can be found in Supporting Information Figure 4. Data shown (mean ± SD) are from one independent experiments with *n* = 10 donors per experiment. Statistical analysis used: Wilcoxon matched‐pairs signed rank test.

Only few proteins were expressed differentially by these populations in an apparently biologically relevant manner, being: HLA‐DR, a class II MHC protein and CD151, a tetraspanin, both markers of T‐cell activation; CD28 and ICOS, both costimulatory receptors and CCR5, a C‐C chemokine receptor, all molecules that are expressed to a much greater extent by the Ki‐67^+^ cells (Fig. 3A, B and Supporting Information Fig. 4). As such, with the exception of these molecules, Ki‐67‐expressing and Ki‐67‐negative EECs are highly similar. Importantly, both subpopulations contain a high degree of granzyme B, but also express T‐bet, CX_3_CR1, perforin, and granulysin (Fig. 3A and Supporting Information Fig. 4, see also Fig. 1B and C), all molecules associated with cytotoxic effector and effector‐memory T cells 17, 18, 22.

### IL‐7Rα^hi^KLRG1^lo^ cells with noncytotoxic memory traits are enriched in lymph nodes

Previously, Sallusto et al. described how CD8^+^ T cells can be distinguished according to CCR7‐expression, establishing the central‐memory (i.e. LN‐homing) and effector‐memory (peripherally active) subset dichotomy 19. Since we found a clear predominance of CCR7 expression by the IL‐7Rα^hi^KLRG1^lo^ MPEC population (Fig. 1B), we were interested in the enumerations of the IL‐7Rα/KLRG1‐defined subsets in human LNs compared to the circulation. For this purpose, we isolated CD8^+^ T‐cell populations from peri‐aortal LN samples acquired from renal transplant recipients (RTRs) just before implantation of the transplant.

**Figure 4:**
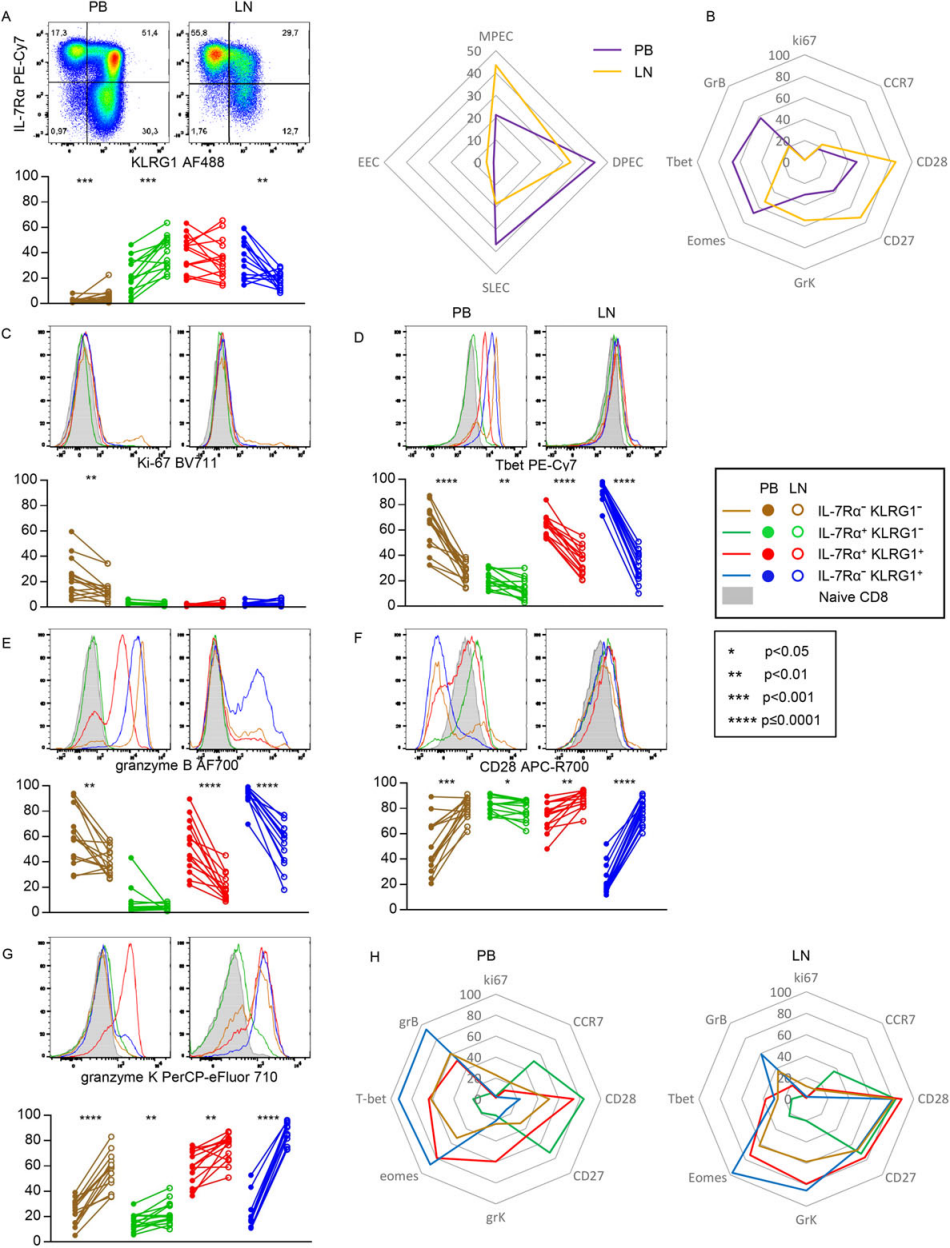
IL‐7Rα^hi^KLRG1^lo^ cells with noncytotoxic memory traits are enriched in lymph nodes. (A) Flow cytometric analysis of the expression of KLRG1 and IL‐7Rα in nonnaive CD8^+^ T cells in peripheral blood (PB; left) and (right), distribution of KLRG1/IL‐7Rα‐defined subsets in nonnaive CD8^+^ T cells of 14 PB with paired LN samples (below) and spider plot of mean (right). (B) Spider plot of mean expression of Ki67, CCR7, CD28, CD27, granzyme K, eomes, T‐bet, and granzyme B in nonnaive CD8^+^ T cells of 14 PB with paired LN samples. (C–G) Representative flow cytometry histograms (top) and frequencies of cells expressing Ki‐67 (C), T‐bet (D), granzyme B (E), CD28 (F), and granzyme K (G) on KLRG1/IL‐7Rα‐defined subsets in nonnaive CD8^+^ T cells of 14 PB with paired LN samples. (H) Spider plot of the mean expression of markers shown in C‐G on PB (left) and LN (right). Data shown (mean ± SD) are representative of three independent experiments with *n* = 2, 7, and 5 donors per experiment. Statistical analysis used: Wilcoxon matched‐pairs signed rank test. CCR7, CD27, and Eomes expression can be found in Supporting Information Figure 6.

Importantly, the composition of the circulating pool of these patients was comparable to that of the healthy individuals (Fig. 1A and Fig. 4A). It was also apparent that IL‐7Rα^hi^KLRG1^lo^ MPECs predominated in LNs as compared to DPECs and SLECs that predominate in the circulation (Fig. 4A). EECs were also detected in low frequencies in the LNs, again uniquely holding a Ki‐67 expressing subpopulation (Fig. 4A, B, and C). KLRG1‐expressing cells were much less often detected in LNs.

In lymph nodes, IL‐7Rα/KLRG1 expression patterns also separated functionally distinct sets of cells (Fig. 4B–H and Supporting Information Fig. 6). However, we found differences between lymph nodes and peripheral blood. Notably, the expression of T‐bet was lower in all subsets retrieved from the lymph nodes, including those of the IL‐7Rα^hi^KLRG1^hi^ DPECs and IL‐7Rα^lo^KLRG1^hi^ SLECS, which was paralleled by a lower expression of granzyme B (Fig. 4D and E). In contrast, CD28 and granzyme K expression was higher by all the non‐MPEC LN subsets when compared to the circulatory subsets (Fig. 4F and G).

### The IL‐7Rα/KLRG1 division defines functionally distinct pools of herpes‐virus specific CD8^+^ T cells

The finding that healthy individuals have high numbers of circulating CD8^+^ T cells with an IL‐7Rα^lo^KLRG1^hi^ SLEC phenotype could mean that they have encountered viruses to which an immune response was recently mounted, or rather, that these populations have arisen as a consequence of persistent infections, such as herpes viruses. To address these distinct options, we used the IL‐7Rα/KLRG1 division to analyse hCMV‐ and EBV‐specific CD8^+^ T cells.

**Figure 5:**
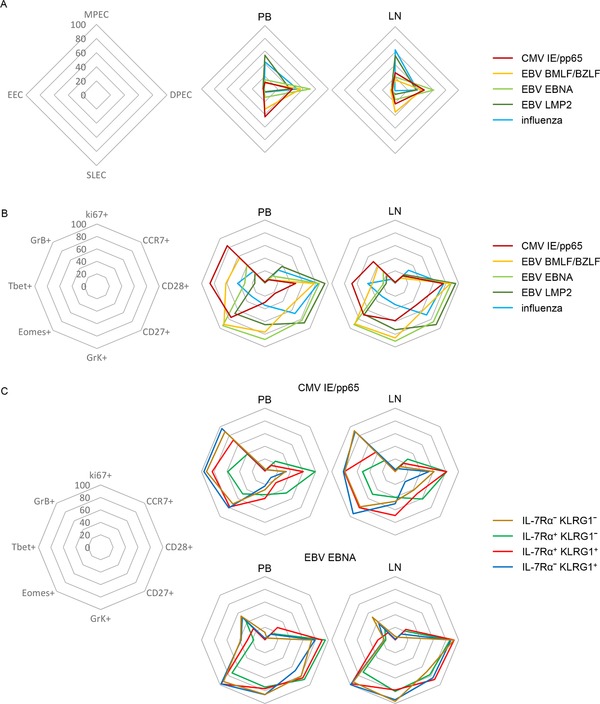
The IL‐7Rα/KLRG1 division defines functionally distinct pools of virus specific CD8^+^ T cells. Flow cytometric analysis of PB and paired LN derived CMV‐IE/pp65 specific CD8^+^ T cells (*n* = 24), EBV BMLF/BZLF‐specific CD8^+^ T cells (*n* = 17), EBV EBNA‐specific CD8^+^ T cells (*n* = 8), EBV LMP2‐specific CD8 T cells (*n* = 13) and influenza‐specific CD8^+^ T cells (*n* = 8); left: layout of spider plot with values, middle: PB, right LN. (A) Spider plots of the distribution of KLRG1/IL‐7Rα‐defined subsets ((mean) examples and individual plots can be found in Supporting Information Fig. 8)). (B) Spider plots of the overall mean percentage of expression of Ki67, CCR7, CD28, CD27, granzyme K, Eomes, Tbet, and granzyme B. (C) Spider plots of the mean percentage of expression of Ki67, CCR7, CD28, CD27, granzyme K, eomes, Tbet, and granzyme B on KLRG1/IL‐7Rα‐defined subsets in nonnaive CD8^+^ T cells (plots for CMV‐IE/pp65 and EBV EBNA are shown, EBV BMLF/BZLF, EBV LMP2, and influenza plots, representative dot plots and individual values can be found in S8). Data shown (mean ± SD) are pooled from three independent experiments with *n* = 2, 7, and 5 donors per experiment.

In RTRs and healthy individuals we found that both circulatory hCMV‐ and EBV BLZF1/BMLF‐specific cells predominantly displayed an IL‐7Rα^hi^KLRG1^hi^ DPECor IL‐7Rα^lo^KLRG1^hi^ SLEC phenotype (Fig. 5A, Supporting Information Figs. 7 (HI) and 8 (RTR)). For all hCMV and lytic EBV (BMLF/BZLF) epitopes, a low percentage of IL‐7Rα^hi^KLRG1^lo^ MPEC phenotype cells was found. In stark contrast, analysis of influenza‐specific CD8^+^ T cells, as an example of CD8^+^ T cells directed against viruses that are effectively cleared by the immune system, revealed that virtually all cells expressed IL‐7Rα, including a significant IL‐7Rα^hi^KLRG1^lo^ MPEC population (Fig. 5A, Supporting Information Figs. 7 (HI) and 8 (RTR)). When further phenotyping these virus‐specific memory populations, we noticed that the expression of molecules associated with a distinct functional profile (Fig. 5B, Supporting Information Figs. 7 and 8). It must be emphasized that virtually all virus‐specific CD8^+^ T cells were Ki‐67‐negative (Fig. 5B, Supporting Information Figs. 7 and 8). Differences on the virus‐specific level between circulating T cells and T cells in the lymph nodes, with regard to T‐bet and granzyme B expression, were as expected, given the findings presented above (Figs. 4, 5B and Supporting Information Fig. 8). However, each herpes virus‐specific population displayed a similar IL‐7Rα/KLRG1 subset distribution in the circulation as in the lymph nodes. Furthermore, similar to what was seen on nonantigen‐specific level, also on the virus‐specific level, decreased levels of T‐bet and granzyme B are present in the LN, regardless of IL‐7Rα/KLRG1 phenotype (Fig. 5C).

### Signatures of hCMV‐ and EBV‐specific CD8^+^ T cells arise during primary infection

**Figure 6:**
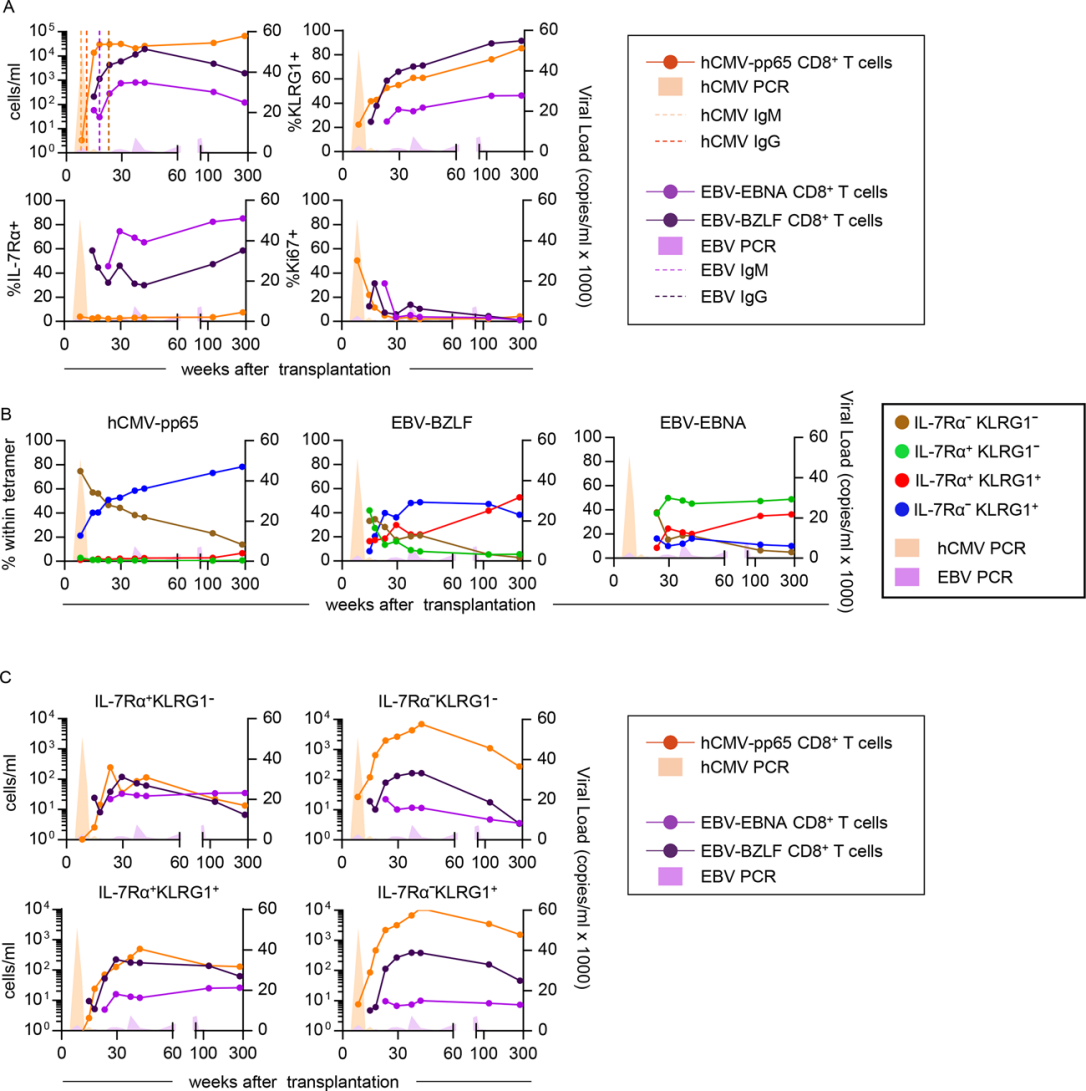
Dynamics and phenotype of hCMV‐ and EBV‐specific CD8^+^ T cells following primary hCMV and EBV in patient 1. (A) Flow cytometric analysis of absolute numbers and frequencies of KLRG1‐expressing, IL‐7Rα‐expressing and Ki‐67‐expressing virus‐specific CD8^+^ T cells from PMBCs. (B) Percentages of KLRG1/IL‐7Rα‐defined subsets within the hCMV‐, EBV‐BZLF‐, and EBV‐EBNA‐specific CD8^+^ T cells. (C) Absolute numbers of KLRG1/IL‐7Rα‐defined subsets in hCMV‐, EBV‐BZLF‐, and EBV‐EBNA‐specific CD8^+^ T cells. Data shown are ten time points from one patient measured in one independent experiment.

The different traits of hCMV and EBV‐specific CD8^+^ T cells, as well as the functional properties of the cells with an SLEC phenotype found during viral latency, urged us to analyse whether virus‐specific T cells found early in the primary antiviral response also differ with respect to their IL‐7Rα/KLRG1 expression pattern and functional traits. To this end, we followed three RTRs experiencing primary hCMV and/or EBV infection in the first year of transplantation. These patients were included during the preprophylaxis treatment era. Consequently, hCMV viral loads were detectable early after transplantation. Since these patients developed no significant hCMV disease, no antiviral treatment was administered during follow‐up. Immunological control, as defined by the absence of detectable viral loads and the appearance of virus‐specific IgG antibodies, was achieved for hCMV within the three months after transplantation and ∼three months later for EBV (Fig. 6A, Supporting Information Figs. 9A, and 10A).

Human CMV and EBV BZLF1/BMLF population dynamics were very much alike with regard to both bigger T‐cell population sizes, as well as a predominance of KLRG1 expressing cells, when compared to EBV EBNA‐specific populations. In contrast EBV EBNA‐specific cells were much more often expressing IL‐7Rα (Fig. 6A and Supporting Information Figs. 9A). Consistent with an early effector function reported in mice, IL‐7Rα^lo^KLRG1^lo^ EEC cells, regardless of specificity, were present initially in the response but generally became a minor fraction after longer follow‐up (Fig. 6B, Supporting Information Figs. 7B and 8B). The hCMV‐specific response was characterized from early on by an abundance of IL‐7Rα^lo^KLRG1^hi^ SLEC cells (Fig. 6B, Supporting Information Figs. 9B and 10B). This was markedly different from the EBV‐specific populations that contained more IL‐7Rα‐expressing cells that were not always coexpressing KLRG1 (Fig. 6B and C, Supporting Information Fig. 9B and C).

**Figure 7:**
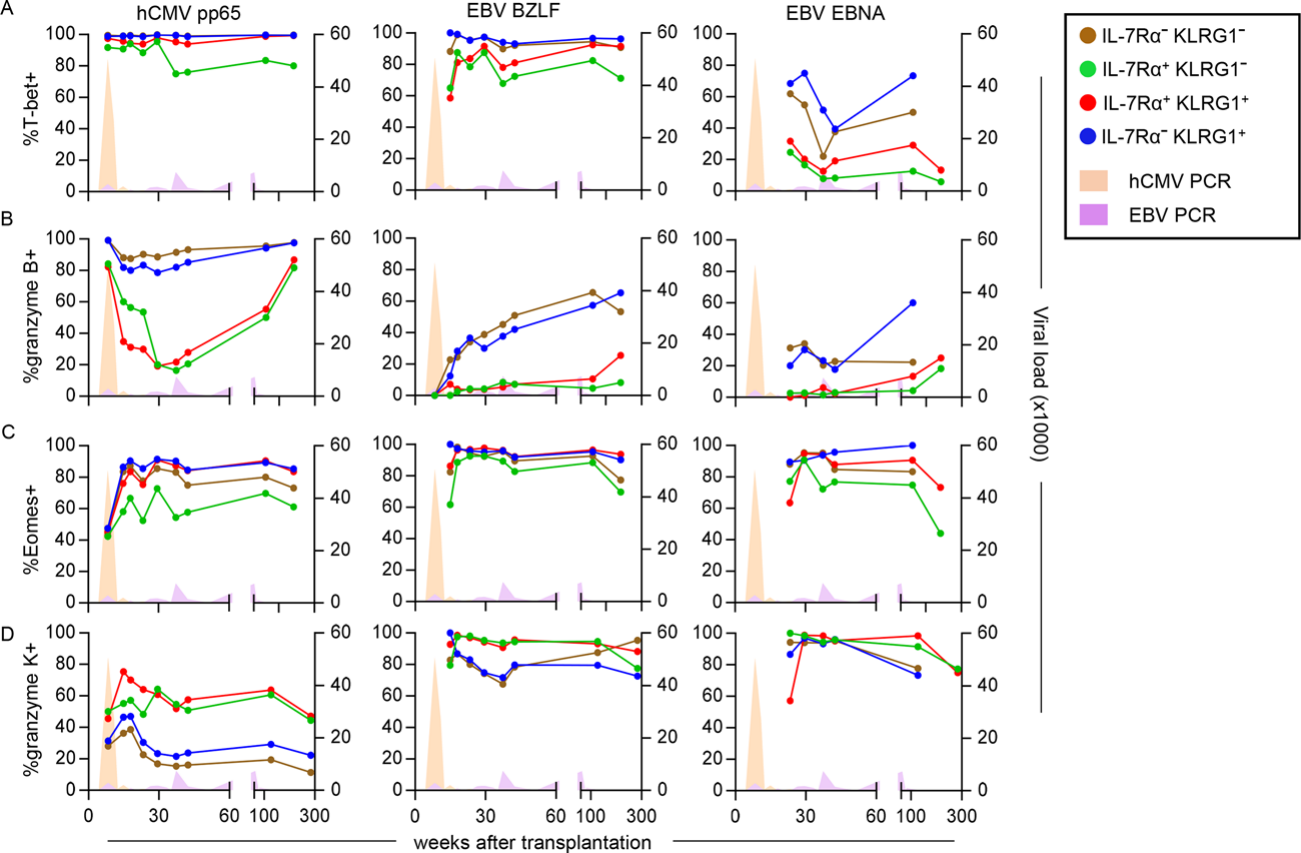
Phenotype of the KLRG1 and IL‐7Rα defined subsets within hCMV‐ and EBV‐specific CD8^+^ T cells following primary hCMV and EBV in patient 1. (A–D) Flow cytometric analysis of the frequencies of cells expressing (A) T‐bet, (B) granzyme B, (C) Eomes, and (D) granzyme K within the KLRG1 and IL‐7Rα defined subsets in hCMV‐, EBV‐BZLF‐, and EBV‐EBNA‐specific CD8^+^ T cells from the PBMCs of patient 1. Data shown are ten time points from one patient measured in one independent experiment.

When looking at the functional traits of the IL‐7Rα/KLRG1 defined subsets within the herpes virus‐specific pools, it became evident that the functional separation found during latency appears to be installed very early in the response. Specifically, early hCMV responses were dominated by CD8^+^ T cells with an EEC or SLEC phenotype abundantly expressing T‐bet and granzyme B (Fig. 6B, 7A, B, Supporting Information Figs. 9–15), whereas EBV‐specific pools hardly contained high T‐bet and granzyme B expressing cells (Fig. 7A, B, Supporting Information Figs. 11–13). Curiously, hCMV‐specific MPECs also expressed considerable amounts of T‐bet and granzyme B, which is unexpected considering the generally infrequent expression of these molecules by MPECs detected during viral latency (Fig. 7A, B, Supporting Information Figs. 11–13). Indeed, expression of T‐bet and granzyme B by MPECs drops markedly as time progresses and patients enter the latency phase. A similar finding concerned Eomes and granzyme K, which was expressed at really highly level already at the earliest time point by EBV‐specific populations, regardless of the IL‐7Rα/KLRG1 phenotype (Fig. 7C, D, Supporting Information Figs. 11–13).

## Discussion

In conclusion, we found that a subset definition based on IL‐7Rα/KLRG1 expression can be used to separate functional sets of (virus‐specific) CD8^+^ T cells in humans during viral latency. From a functional point of view, the definition derived from the mouse data appears to be largely in accord with the findings in humans. IL‐7Rα^hi^KLRG1^lo^ (in mice defined as MPEC) concern noncytotoxic cells with memory functions, having high levels of CCR7 and low expression of T‐bet and granzyme B. Remarkably, the expression of Eomes, a factor strongly implicated in memory cell generation in mice, is relatively low in these cells and in these anatomical compartments. In contrast, IL‐7Rα^lo^KLRG1^hi^ cells (in mice defined as SLEC) found during latency strongly resemble cytotoxic effector‐type cells, abundantly expressing both T‐bet and granzyme B. It is however unlikely that human IL‐7Rα^lo^KLRG1^hi^ cells found during latency are short‐lived as initially was described for their murine counterparts. None of the latently infected subjects analysed had clinical or laboratory signs of viral replication. Further, in accordance with the absence of antigen‐driven proliferative T‐cell responses, no Ki‐67 expression was found in the IL‐7Rα^lo^KLRG1^hi^ pool. Finally, studies analysing the turn‐over of T‐cell subsets in humans have shown that the population of cells with constitutive effector functions actually has a long lifespan 23, 24. Therefore, we argue that to coin the term SLEC to human CD8^+^ T cells with an IL‐7Rα^lo^KLRG1^hi^ phenotype would be a misnomer. Rather, the expression of KLRG1 on human CD8^+^ T cells appears to be associated with the acquisition of immediate effector functions during acute and latent viral infection. In fact, also in the mouse antigen‐specific IL‐7Rα^lo^KLRG1^hi^ CD8^+^ T cells can be detected long‐time after infection 25, 26.

IL‐7Rα^lo^KLRG1^lo^ cells (in mice defined as EEC) are similar to the IL‐7Rα^lo^KLRG1^hi^ cells with the big difference that this population holds a subset of activated and actively cycling cells. Nevertheless, by their expression of CCR7 and CCR4, they also resemble the noncytotoxic IL‐7Rα^hi^KLRG1^lo^ MPECs. We therefore hypothesize that these cells concern recently activated naïve or MPEC cells that have lost expression of IL‐7Rα while having acquired expression of typical effector molecules. Future investigations should focus on their functional role in maintaining immunological control over latent viral infections. The finding of substantial populations of IL‐7Rα^hi^KLRG1^hi^ DPECs in healthy individuals is also interesting. These cells express large amounts of granzyme K and granzyme B but are still susceptible to IL‐7R‐mediated signalling, a feature that is normally associated with maintenance of noncytotoxic memory populations in mice 27. Also here, further investigations are indicated into their functional role, especially in controlling EBV infection as the EBV‐specific CD8^+^ T cells harbour particularly large amounts of these cells.

Additionally, analyses of IL‐7Rα/KLRG1 expression allows for a clear separation of functionally different human CD8+ T‐cell subsets also in LNs. In line with the previous findings of Sallusto et al. 19, CCR7‐expressing cells, mainly displaying an IL‐7Rα^hi^KLRG1^lo^ phenotype, predominate in this anatomical compartment. However, the distinctions between noncytotoxic (memory) and cytotoxic (effector) traits are less apparent in the LNs, since typical effector traits are expressed significantly less often in this environment than in the circulation, even by the KLRG1‐expressing subsets. These findings also hold true on the antigen (virus)‐specific level and the analyses we performed here reveal a number of additional specific features of virus‐specific cells. Depending on the anatomical location and on the anti‐viral specificity, the distribution of the IL‐7Rα/KLRG1‐defined subsets differs markedly. We regard it likely that the differences between virus‐specific populations with regard to IL‐7Rα/KLRG1 expression and phenotype and function in a general sense, are the result of a complex combination of signals (e.g. as transduced via the T‐cell receptor, costimulatory and coinhibitory receptors, and cytokine receptor interactions) that is delivered to the T cells in a certain spatiotemporal context that is distinct for each viral infection. This may not be so difficult to understand given the fact that each virus exhibits its own specific mode of infection, primarily targeting different organ systems/cell types, were some viruses are cleared after infection while others establish latency. Even immunomodulatory viral aspects, such as the hCMV‐mediated downregulation of MHC class I molecules may be involved in this process 28. Also on the virus‐specific level, KLRG1‐expressing cells are largely excluded from lymph nodes and therefore may function primarily at nonlymphoid compartments. As our studies in the primary infection showed that, already early in the response, distinct viruses elicit differential T‐cell responses and analyses of the T cells during acute infection might provide early qualitative information on the T‐cell response that provides protection during the latency phase. This insight is of particular importance to the creation of effective vaccination strategies in the future where one would aim to establish a functionally distinct memory T‐cell population that provides protection against a pathogen on the long term.

## Materials and methods

### Subjects

In order to study the dynamics and phenotype of acute phase hCMV‐ and EBV‐specific CD8^+^ T cells compared to those present during the latency phase, hCMV‐specific CD8^+^ T cells from 2 hCMV‐ and EBV‐seronegative recipients of a hCMV‐ and EBV‐seropositive kidney transplant were analyzed longitudinally (Table 1, patient 1–2). To eliminate the influence of a primary EBV infection on the hCMV‐specific CD8^+^ T cells we also studied the hCMV‐specific CD8^+^ T cells from one kidney transplant recipient who was hCMV‐seronegative prior to transplantation and received a hCMV‐seropositive kidney (Table 1, patient 3). The treatment of each patient as well as other relevant information can be found in Table 1.

**Table 1:** Patient characteristics primary infections

				Tetramers used				
Patient	Age	HLA‐I typing	Primary infection	hCMV pp65	IE	EBV BZLF	EBNA	Immunosuppressive Regimen*
1	24	A1/A3 B7/B35	hCMV/EBV	TPR		EPL	RPP	CsA/P/MMF
2	25	A3 B35	hCMV/EBV	IPS		EPL	HPV	CsA/P/MMF
3	58	A2/A3 B7/B35	hCMV	TPR	VLE			FK/P/MMF/CD25 mAb

^*^CsA, cyclosporine A; P, Prednisolon; MMF, mycophenolate mofetil; CD25mAb, Basilixumab induction therapy; FK, Tacrolimus.

Latent phase peripheral blood (PB) and LN hCMV‐, EBV‐, and influenza‐specific CD8^+^ T cells were analyzed in hCMV‐ and EBV‐seropositive renal transplant recipients (*n* = 8; Table 2). HCMV‐, EBV‐, and influenza‐specific CD8^+^ T cells were also analyzed in EBV and CMV serotyped healthy individuals (*n* = 12, Table 3). Further analysis of KLRG1/IL7Rα‐defined subsets in total nonnaïve CD8^+^CD3^+^ was performed on PBMC derived from buffy coats (*n* = 10).

**Table 2:** Kidney transplant recipients (PB and LN): Tetramers analyzed and EBV/CMV serostatus

Patient	CMV IE/pp65	EBV BMLF/BZLF	EBV EBNA	EBV LMP2	Influenza	EBV	CMV
1221	IPS / NLV / VLE	EPL / GLC	HPV	CLG / FLY		+	+
1257	YSE / ELK / ELR / QIK	RAK	FLR	CLG		+	+
1276	YSE / ELR / TPR	RAK	FLR		CTE	+	+
1332	NLV	GLC		FLY		+	+
1338	NLV	GLC		FLY	GIL	+	+
1384	YSE / NLV / QIK / ELR / ELK	GLC / RAK	FLR	FLY / CLG	GIL / CTE	+	+
1404	NLV	GLC		FLY / CLG	GIL	+	+
1442	NLV	GLC		FLY / CLG		+	+
1448	NLV	GLC		FLY	GIL	+	+
1461	TPR	RAK	FLR		CTE	+	+
1510		GLC		FLY / CLG	GIL	+	–
1532	YSE / IPS / ELR	RAK / EPL	HPV / FLR			+	+
1683		GLC / RAK	FLR	FLY / CLG		+	–

**Table 3:** Healthy donors: Tetramers analysed and EBV/CMV serostatus

	CMV IE/pp65	EBV BMLF/BZLF	EBV EBNA	EBV LMP2	Influenza	EBV	CMV
H1	NLV	GLC		FLY	GIL	+	+
H2	NLV / VLE	GLC		CLG		+	+
H3	NLV / VLE / QIK / ELR / ELK	GLC / RAK	FLR	FLY	GIL	+	+
H4		RAK	FLR / RPP			+	–
H5	TPR	GLC / RAK	FLR	FLY / CLG		+	+
H6	NLV / TPR / TPR / VLE	GLC	RPP	FLY / CLG	GIL	+	+
H7	NLV / IPS	GLC / EPL	HPV	FLY / CLG	GIL	+	+
H8		GLC / EPL	HPV / RLR / RPP	FLY / CLG	GIL	+	–
H9	NLV / TPR	GLC			GIL	+	+
H10	NLV	GLC		CLG	GIL	+	+
H11		GLC / RAK	FLR / RLR	FLY / CLG		+	–
H12	YSE / ELK	RAK	FLR		CTE	+	+

PBMCs were isolated prior to transplantation and lymph node mononuclear cells (LNMC) were isolated during kidney transplantation. All patients were treated with quadruple immunosuppression, consisting of CD25mAb induction therapy, Prednisolone, calcineurin‐inhibitor, and mycophenolic acid. Except for CD25mAb, immunosuppressive treatment was started after transplantation. At the time the lymph node was gathered the first dose of CD25mAb was already administered. However, we have demonstrated that CD25mAb (basiliximab, Novartis Pharma, Basel, Switzerland) was not detectable in LNMC and that ex vivo CD25 expression on LNMC could be blocked with CD25mAb 29.

### Ethics statement

The medical ethics committee of the Academic Medical Center, Amsterdam, approved the study and all subjects gave written informed consent.

### Isolation of mononuclear cells from peripheral blood and lymph nodes

PBMCs were isolated using standard density gradient centrifugation. Lymph nodes were collected from kidney transplant recipients during living donor kidney transplantation. Briefly, LNMCs were isolated from surgical residual material of the recipient, gathered during the implantation of the transplanted kidney. Before anastomizing the arteria and vena renalis, the iliac artery and vein are dissected free. The residual tissue removed in this procedure, to obtain adequate vascular exposure, often contains lymph nodes. Directly after extraction, the gathered lymph nodes were chopped in small pieces. A cell suspension was obtained by grinding the material through a flow‐through chamber. PBMCs and LNMCs were subsequently cryopreserved until the day of analysis.

### Virological analysis

Quantitative PCR for hCMV was performed in EDTA (ethylenediaminetetraacetic acid) whole blood samples, as described (2). To determine CMV serostatus, anti‐CMV immunoglobulinG (IgG) was measured in serum using the AxSYM microparticleenzyme immunoassay (Abbott Laboratories, Abbott Park, IL, USA) according to the manufacturer's instructions. Measurements were calibrated relative to a standard serum. EBV serostatus was determined by qualitative measurement of specific IgG against the viral capsid antigen (VCA) and against nuclear antigen of EBV using respectively the anti‐EBV VCA IgG ELISA and the anti‐EBV nuclear antigen of EBV IgG ELISA (Biotest, Dreieich, Germany). All tests were performed following the instructions of the manufacturers.

### Immunofluorescence staining, flowcytometry

Mononuclear cells were washed in PBS containing 0.01% (wt/vol) NaN3 and 0.5% (wt/vol) BSA. Two million PBMCs were incubated with APC/PE/BV421 and AF488‐labeled tetrameric‐complexes for either hCMV‐pp65, hCMV‐IE‐1, EBV‐BZLF1, EBV‐BMLF‐1, EBV‐EBNA‐1, EBV‐EBNA‐3a, EBV‐LMP2, and Influenza A virus‐matrix protein 1 (Table 4) (courtesy, The NIH Tetramer Facility, Emory University), followed by incubation with a combination of the following antibodies: CD127 (IL7Rα) PE‐Cy7 (eBioRDR5), CD152 (CTLA4) PerCP‐eFluor 710 (14D3), CX_3_CR1 APC (2A9‐1), HLA‐DR AF700 (LN3), ICOS APC (ISA‐3), KLRG1 PE (13F12F2), KLRG1 PerCP‐eFluor 710 (13F12F2), CD27 APC‐eFluor 780 (O323) (eBioscience, Thermo Fisher Scientific, Breda, Netherlands), CD127 BV650 (A019D5), CD151 PE (50‐6), CD160 PE‐Cy7 (BY55), CD186 (CXCR6) PE‐Cy7 (K041E5), CD194 (CCR4) BV421 (L291H4), CD196 (CCR6) AF488 (G034E3), CD244 (2B4) PE‐Dazzle 594 (C1.7), CD27 APC‐Fire 750 (O323), CD38 BV421 (HIT2), CD45RA APC (HI100), CD57 PE‐Dazzle 594 (HNK‐1), CD8 BV785 (RPA‐T8), CXCL16 PE (22‐19‐12), KLRG1 BV421 (14C2A07) (BioLegend, San Diego, CA, USA), CD94 APC (HP‐3D9), CD28 APC‐R700 (CD28.2), CD279 (PD1) BB515 (EH12.1), CD45RA BUV395 (HI100), CD95 BUV395 (DX2), CD184 (CXCR4) BUV395 (12G5), CD197 (CCR7) BUV395 (150503), CD197 PE‐Cy7 (3D12), CD45RA BV650 (HI100), CD3 BUV496 (UCHT1), CD25 PE (2A3), CD3 V500 (UCHT1) (BD Biosciences, San Jose, CA, USA), CD183 (CXCR3) PE‐Vio615 (REA232) (Miltenyi Biotec B.V. Bergisch Gladbach, Germany) and KLRG1 Alexa Fluor 488 30. Dead cells were excluded from the analysis by using Fixable viability dye eFluor 506 or the Live‐dead fixable RED (Invitrogen, Thermo Fisher Scientific, Breda, the Netherlands).

**Table 4:** Tetrameric complexes used

Name	HLA	Virus	Protein	Peptide	Labels
YSE	HLA‐A*0101	hCMV	pp65	YSEHPTFTSQY	PE
NLV	HLA‐A*0201	hCMV	pp66	NLVPMVATV	BV421
TPR	HLA‐B*0702	hCMV	pp65	TPRVTGGGAM	APC
IPS	HLA‐B*3501	hCMV	pp65	IPSINVHHY	APC
VLE	HLA‐A*0201	hCMV	IE‐1	VLEETSVML	PE
QIK	HLA‐B*0801	hCMV	IE‐1	QIKVRVKMV	PE/APC
ELK	HLA‐B*0801	hCMV	IE‐1	ELKRKMIYM	APC
ELR	HLA‐B*0801	hCMV	IE‐1	ELRRKMMYM	APC
EPL	HLA‐B*3501	EBV	BZLF‐1	EPLPQGQLTAY	AF488
RAK	HLA‐B*0801	EBV	BZLF‐1	RAKFKQLL	BV421
GLC	HLA‐A*0201	EBV	BMLF‐1	GLCTLVAML	APC
HPV	HLA‐B*3501	EBV	EBNA‐1	HPVGEADYFEY	PE
RLR	HLA‐A*0301	EBV	EBNA‐3a	RLREAQVK	BV421
RPP	HLA‐B*0702	EBV	EBNA‐3a	RPPIFIRRL	AF488/BV421
FLR	HLA‐B*0801	EBV	EBNA‐3a	FLRGRAYGL	AF488
CTE	HLA‐A*0101	Influenza A virus	Nucleoprotein	CTELKLSDY	APC
GIL	HLA‐A*0201	Influenza A virus	Matrix Protein‐1	GILGFVFTL	APC/AF488
CLG	HLA‐A*0201	EBV	LMP‐2	CLGGLLTMV	BV421
FLY	HLA‐A*0201	EBV	LMP‐2	FLYALALLL	AF488

The FOX‐p3 staining kit (eBioscience) was used for intracellular stainings with the following antibodies: Granzyme B AF700 (GB11), T‐bet AF647 (O4‐46), Fox‐P1 PE (JC12) (BD Biosciences), Hobit (Sanquin) followed by secondary staining with IgMa FITC (MA‐69), granulysin PE (DH2), Helios Pacific Blue (22F6), Ikaros AF647 (16B5C71), Ki67 BV711 (Ki‐67), perforin BV421 (B‐D48), Tbet BV421 (4B10) (Biolegend), eomesodermin PerCP‐eFluor 710 (WD1928), granzyme K PerCP‐eFluor 710 (G3H69), helios PE‐Cy7 (22F6), T‐bet PE‐Cy7 (eBio4B10), (eBioscience, Thermo Fisher Scientific) and granzyme K PE (Immunotools, Friesoythe, Germany). Measurements were done on a LSR Fortessa flow cytometer (BD) and analysis was performed with FlowJo software (PC version 10) (FlowJo, Ashland, OR, USA).

Gating strategy can be found in Supporting Information Figure 1. All panels used can be found in Supporting Information Table 1. The guidelines for the use of flow cytometry and cell sorting in immunological studies were followed 31.

### RNA‐seq data analysis

CD8^+^ T cells were isolated from buffycoat derived PBMC (from six different donors) with CD8^+^ T‐cell beads (Miltenyi Biotec B.V.). Subsequently they were stained with a‐CD3 eF450 (cloneSK7), a‐CD127 (IL7Rα) PE‐Cy7 (eBioRDR5) (eBioscience), a‐CD8 APC (RPA‐T8), a‐CD45RA PE (HI100) (BD Biosciences), a‐CD27 BV510 (O323) (Biolegend), a‐KLRG1 AF488 (kind gift of HP Pircher) and live/dead fixable near IR (Invitrogen) and sorted into all four KLRG1/IL‐7Rα‐defined CD8^+^CD3^+^ subsets on a FACS ARIA III sorter (BD) (Supporting Information Fig. 3). RNA was isolated with the Qiagen RNeasy Plus Micro kit (Qiagen Benelux B.V., the Netherlands) according to manufacturer's instructions and subsequently sequenced with Illumina NextSeq 500 platform, single‐end protocol with a read length of 76 nt. The reads were aligned to hg38 reference genome, with STAR (v2.4.0j) aligner software using defaults settings. The reads that aligned to (only exonic parts of) each gene were quantified using feature Counts (v1.4.3‐p1) software and Ensembl annotation. After visual inspection of MDS plots and (log2 counts) boxplots of all the samples, in combination of Bioanalyzer RIN scores, we decided to remove six samples. Log2 transformed CPM (counts per million + 0.5) were made in edgeR (v3.14.0), genes with less than 1 CPM in 3 or more samples were removed from further analysis. Subsequently, limma (v3.28.21) trend was used to test for significantly expressed genes between the groups, making use of a block (aka paired) design that takes donor information into account. An FDR‐adjusted *p*‐value < 0.05 was considered significant. Hierarchical clustering was done using only genes that were significant in at least one of six limma comparisons (in total 2890 genes), using z‐scores of log2 transformed RPKM (reads per kilobase per million reads + 0.5) values. Using Ward.D clustering method with Euclidean distance in gplots (v3.0.1), the genes were divided in seven groups. Enrichment analysis of GO terms was done for each group, using clusterProfiler (v3.0.5). Analyses with bioconductor packages were all done in R (v3.3.2). Data are available from the GEO under accession number GSE113098.

### Statistical analysis

The Wilcoxon matched‐pairs signed rank test was used for analysis of differences between paired samples. A *p*‐value of < 0.05 was considered statistically significant.

## Conflict of interest

The authors declare no financial or commercial conflict of interest.

**Abbreviations**
DPEC: double positive effector cell
EEC: early effector cell
Eomes: eomesodermin
HI: healthy individual
IL‐7Rα: IL‐7 receptor‐α
KLRG1: killer cell lectin‐like receptor subfamily G member 1
MPEC: memory precursor effector cell
PB: peripheral blood
RTR: renal transplant recipient
SLEC: short‐lived effector cell

## Supporting information

Supporting Information
