# Supplementary material for: Expression of IL‐7Rα and KLRG1 defines functionally distinct CD8+ T‐cell populations in humans
Source: Eur J Immunol. 2019 Mar 25;49(5):694–708. doi: 10.1002/eji.201847897 (PMC6593687; doi:10.1002/eji.201847897)
Supplement: Supplementary file 1 — Supporting Information [file EJI-49-694-s001.pdf]

European Journal of Immunology

Supporting Information
for
DOI 10.1002/eji.201847897

Ester B. M. Remmerswaal, Pleun Hombrink, Benjamin Nota, Hanspeter Pircher, Ineke J. M. ten Berge, René A. W. van Lier and Michiel C. van Aalderen

Expression of IL-7Rα and KLRG1 defines functionally distinct CD8$^+$ T-cell populations in humans

© 2019 The Authors. *European Journal of Immunology* published by WILEY-VCH Verlag GmbH & Co. KGaA, Weinheim.

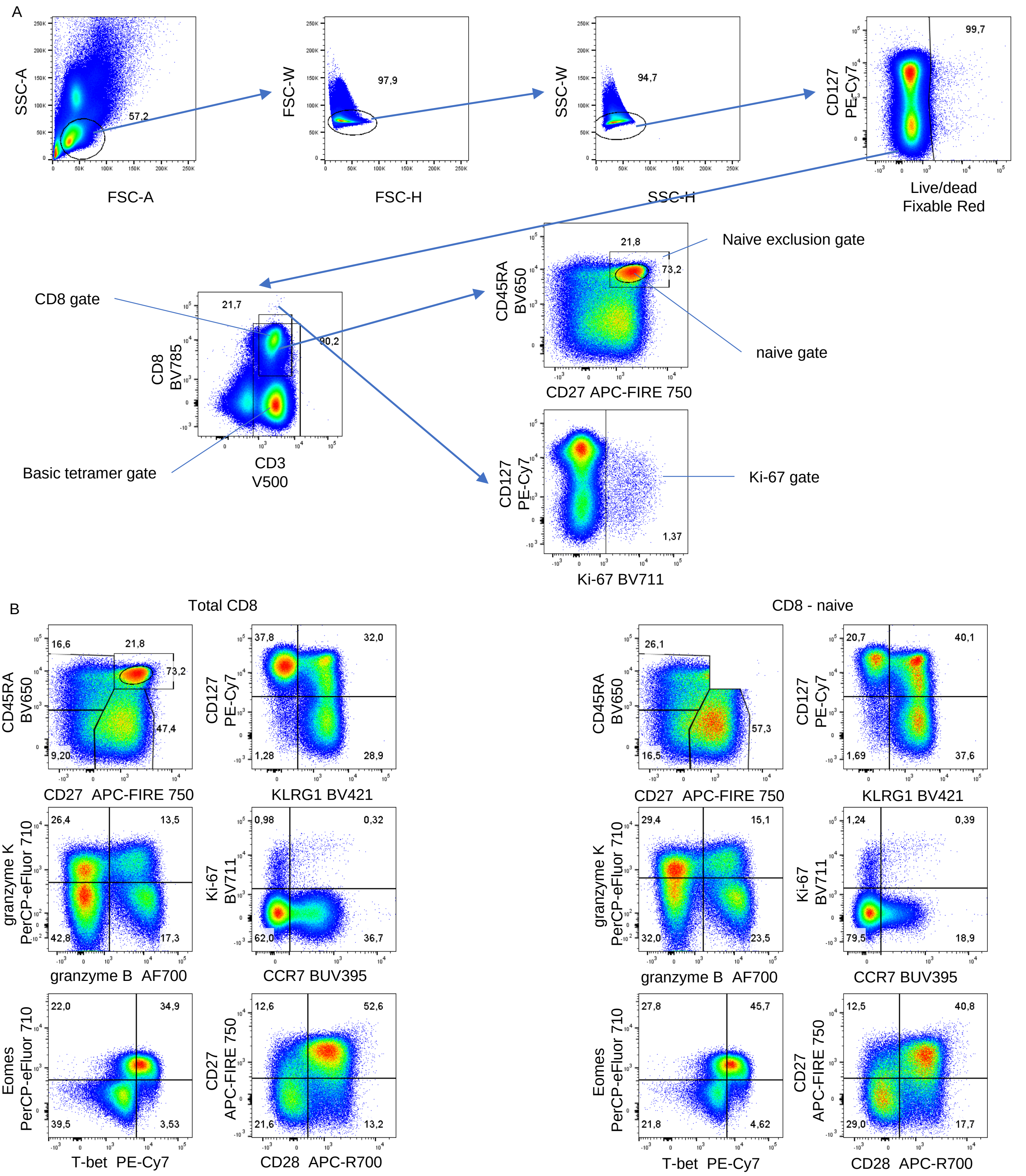

Figure S1. Gating strategy of single live non-naive KLRG1/IL-7Rα-defined CD8$^+$CD3$^+$ T cell subsets and comparison of non-naive CD8$^+$ T cells to total CD8$^+$ T cells.

A) Non-naive CD8$^+$ T cells were analysed by gating single live CD3$^+$CD8$^+$ lymphocytes from which the CD27$^+$CD45RA$^+$ cells were excluded. Tetramer positive cells were analysed by gating single live CD3$^+$ lymphocytes and than plotting CD8 vs tetramer (examples of tetramer stainings: S7 and S8). To calculate foldchanges changes the geomean fluorescence intensity (GMFI) of the naive gate was used as comparison .

B) Comparison of total CD8$^+$ T cells (left) and non-naive CD8$^+$ T cells (right).

fig 1 and S2, 4 and S6, 5 and S7, S8				performed on MNC from 14 paired PB / LN samples and 12 healthy individuals											
panel	BUV395	x	BV421	V500	BV650	BV711	BV785	AF488	PerCP-eFluor 710	PE	PE-Dazzle594	PE-Cy7	APC	APC-R700	APC-FIRE750
1A	CD45RA	x	tetramer	CD3	CD127	Ki67	CD8	KLRG1	Eomes	tetramer	Live-dead fixable RED	T-bet	tetramer	CD28	CD27
1B	CD45RA	x	tetramer	CD3	CD127	Ki67	CD8	tetramer	Eomes	KLRG1	Live-dead fixable RED	T-bet	tetramer	CD28	CD27
1C	CD45RA	x	KLRG1	CD3	CD127	Ki67	CD8	tetramer	Eomes	tetramer	Live-dead fixable RED	T-bet	tetramer	CD28	CD27
panel	BUV395	x	BV421	V500	BV650	BV711	BV785	AF488	PerCP-eFluor 710	PE	PE-Dazzle594	PE-Cy7	APC	AF700	APC-FIRE750
2A	CCR7	x	tetramer	CD3	CD45RA	Ki67	CD8	KLRG1	granzyme K	tetramer	Live-dead fixable RED	CD127	tetramer	granzyme B	CD27
2B	CCR7	x	tetramer	CD3	CD45RA	Ki67	CD8	tetramer	granzyme K	KLRG1	Live-dead fixable RED	CD127	tetramer	granzyme B	CD27
2C	CCR7	x	KLRG1	CD3	CD45RA	Ki67	CD8	tetramer	granzyme K	tetramer	Live-dead fixable RED	CD127	tetramer	granzyme B	CD27

fig 2 and S4				performed on PBMC from 6 buffycoats											
panel	x	x	eF450	BV510	x	x	x	AF488	x	PE	x	PE-Cy7	APC	x	APC-FIRE750
1	x	x	CD3	CD27	x	x	x	KLRG1	x	CD45RA	x	CD127	CD8	x	Live-dead fixable near IR

fig 3 and S3, S5				performed on PBMC from 10 buffycoats											
panel	BUV395	BUV496	BV421	eFluor506	BV650	BV711	BV785	AF488<br>BB515<br>FITC	PerCP-eFluor 710<br>PerCP-Cy5.5	PE	PE-Dazzle594	PE-Cy7	APC	APC-R700	APC-FIRE750
1	CD95	CD3	Perforin (B-D48)	Fixable viability dye	CD45RA	Ki67	CD8	KLRG1	granzyme K	granulysin	CD57	CD127	CX3CR1	granzyme B	CD27
2	CD45RA	CD3	KLRG1	Fixable viability dye	CD127	Ki67	CD8	Hobit	Eomes	FOX-P1	x	Helios	T-bet	CD28	CD27
3	CD45RA	CD3	CD38	Fixable viability dye	CD127	Ki67	CD8	KLRG1	CTLA4	CD25	x	CCR7	ICOS	HLA-DR	CD27
4	CD45RA	CD3	KLRG1	Fixable viability dye	CD127	Ki67	CD8	PD1	x	CXCL16	CD244	CD160	Ikaros	x	CD27
5	CXCR4	CD3	CCR4	Fixable viability dye	CD127	Ki67	CD8	CCR6	KLRG1	CD151	CXCR3	CXCR6	CD45RA	x	CD27

fig 6 and 7, S9, S10, S11, S12, S13, S14				performed on PBMC from 10, 14 and 9 timepoints from renal transplant recipients following transplantation											
panel	x	x	BV421	V500	BV650	BV711	BV785	AF488	PerCP-eFluor 710	PE	PE-Dazzle594	PE-Cy7	APC	AF700	APC-eFluor780
1	x	x	T-bet	CD3	CD45RA	Ki67	CD8	KLRG1	Eomes	granzyme K	Live-dead fixable RED	CD127	tetramer	granzyme B	CD27

intracellular
surface
tetramer

Table s1: panels per figure.
Overview of the combination of monoclonals/fluorochromes used per panel per figure.

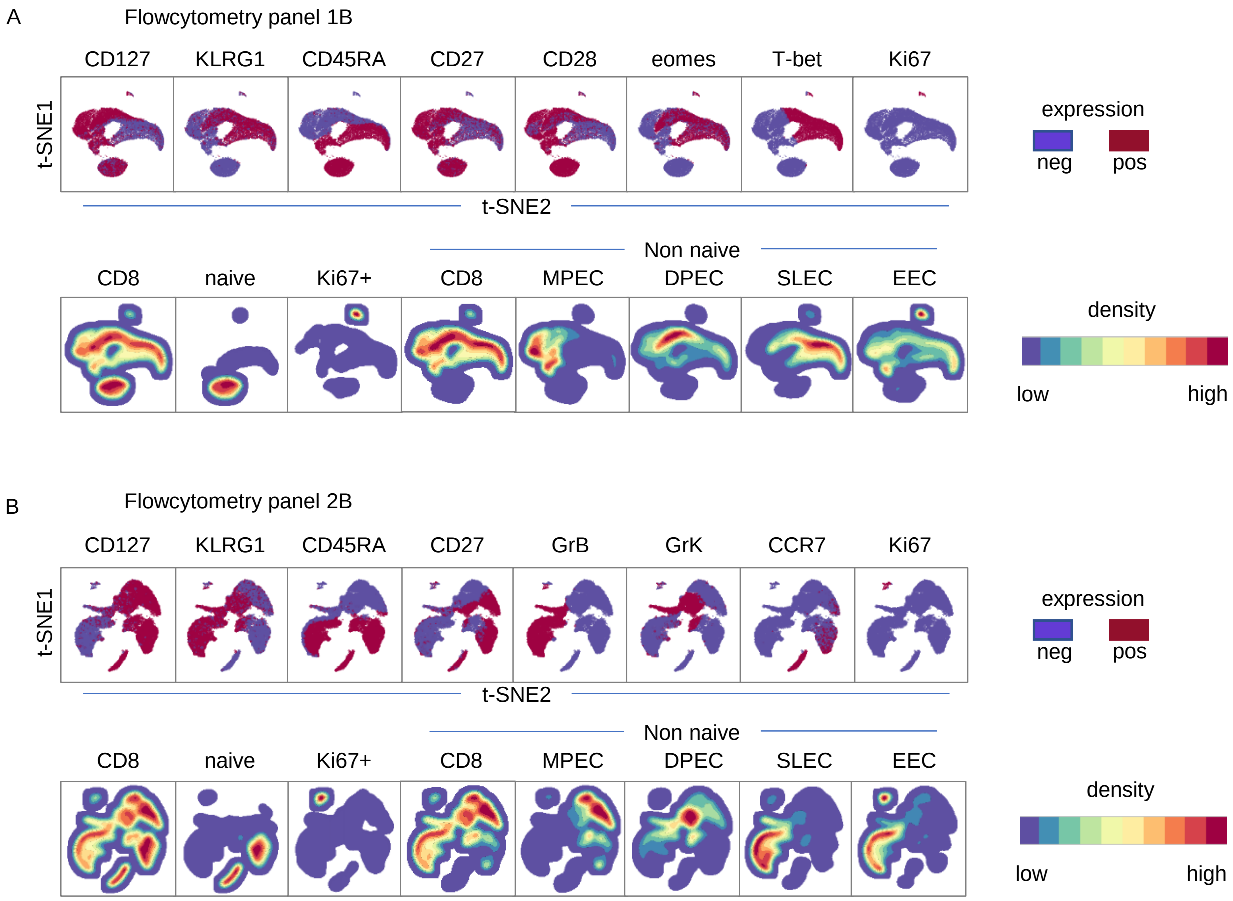

Figure S2. H-SNE analysis of non-naive KLRG1/IL-7Rα-defined $CD8^+CD3^+$ T cell subsets from figure 1 with Cytosplore[1,2].

All compensated parameters of total live $CD8^+CD3^+$, naive $CD8^+CD3^+$, non-naive $CD8^+CD3^+$ cells and the non-naive KLRG1/CD127-defined subsets from 12 unique healthy donor PBMC were exported from FlowJo (v10) and analysed in Cytosplore (v4.5.0 - build 22.20.16.4836) with H-SNE.

Acitve parameters for H-SNE were

A) CD45RA, CD27, CD28, Eomes, T-bet and Ki-67 (table S1: panel 1B)

B) CD45RA, CD27, granzyme B, granzyme K, CCR7 and Ki-67. (table S1: panel 2B)

IL-7Rα and KLRG1 were not used as active parameters in the H-SNE in either analysis, but only analysed for their expression. All settings were left as automatically set by Cytosplore. Number of scales=5.

A-B) Upper row: expression of markers. Lower row: clusterdensity of total, naive, ki-67-expressing, non-naive $CD8^+CD3^+$ T cells and non-naive KLRG1/CD127-defined $CD8^+CD3^+$ T cell subsets (left to right).

Data shown (mean +-SD) are pooled from/representative of 3 independent experiments with n=8, 2 and 4 donors per experiment.

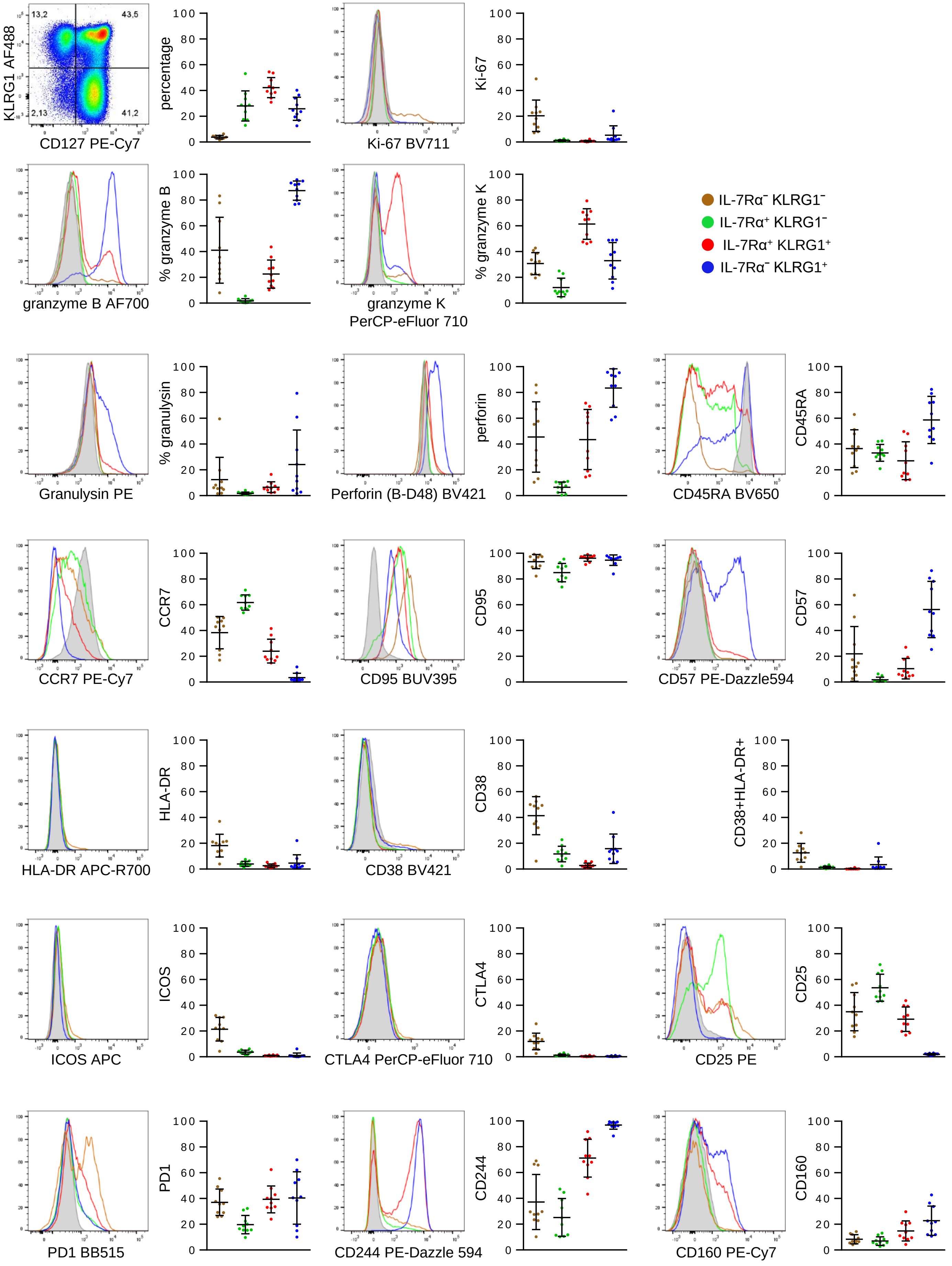

Figure S3. Phenotype of non-naive KLRG1/IL-7Rα-defined CD8⁺CD3⁺ T cell subsets. (continued on next page)

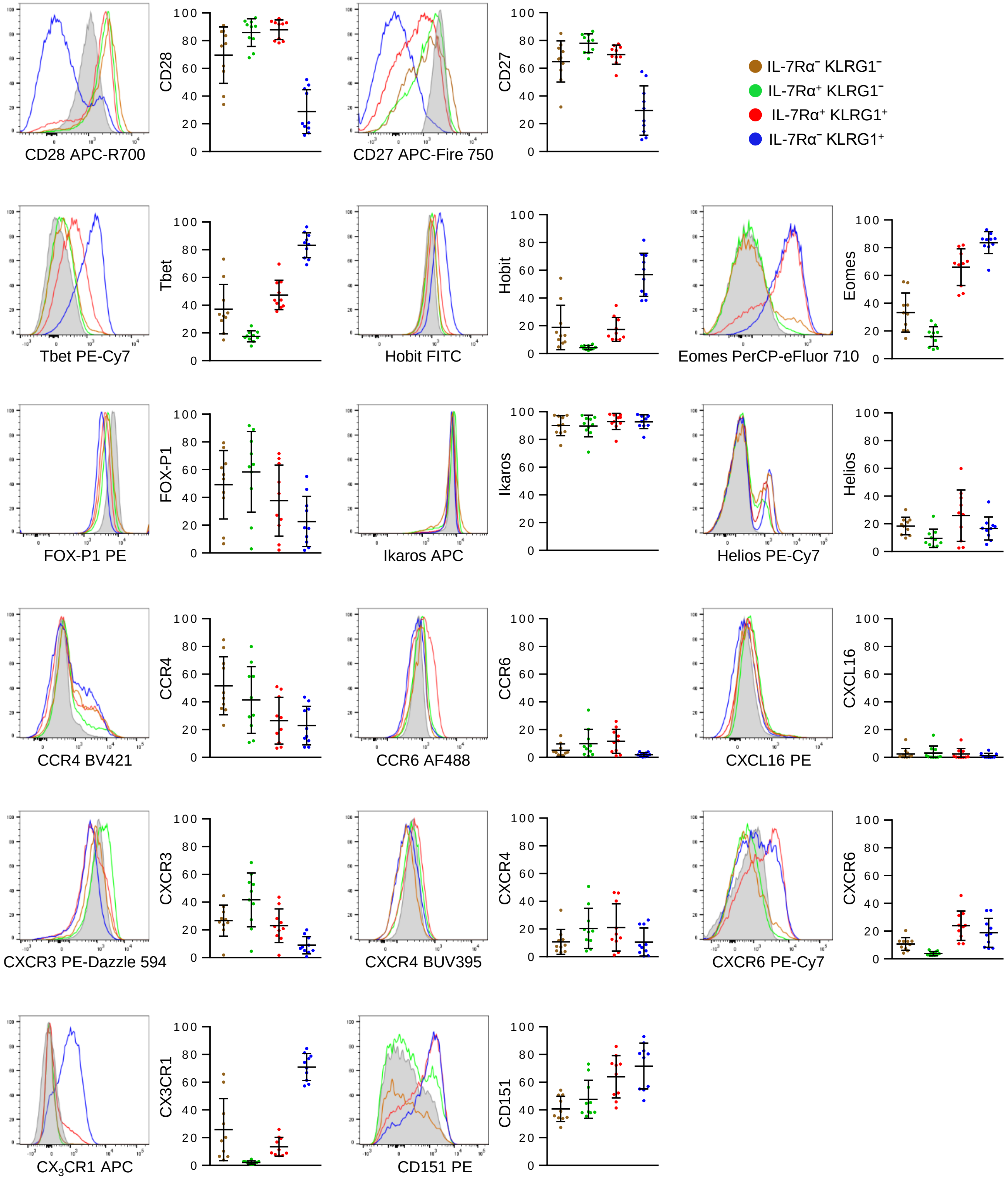

Figure S3 continued. Phenotype of non-naive KLRG1/IL-7Rα-defined $CD8^+CD3^+$ T cell subsets.
Representative histograms and graphs of the individual percentages of expression on each of the four KLRG1/IL-7Rα-defined subsets in non-naive $CD8^+$ T cells of 10 buffy coats compared to the naïve ($CD27^+CD45RA^+$) $CD8^+$ T cells of the same sample.
Data shown (mean +-SD) are pooled from 1 independent experiments with n=10 donors per experiment.

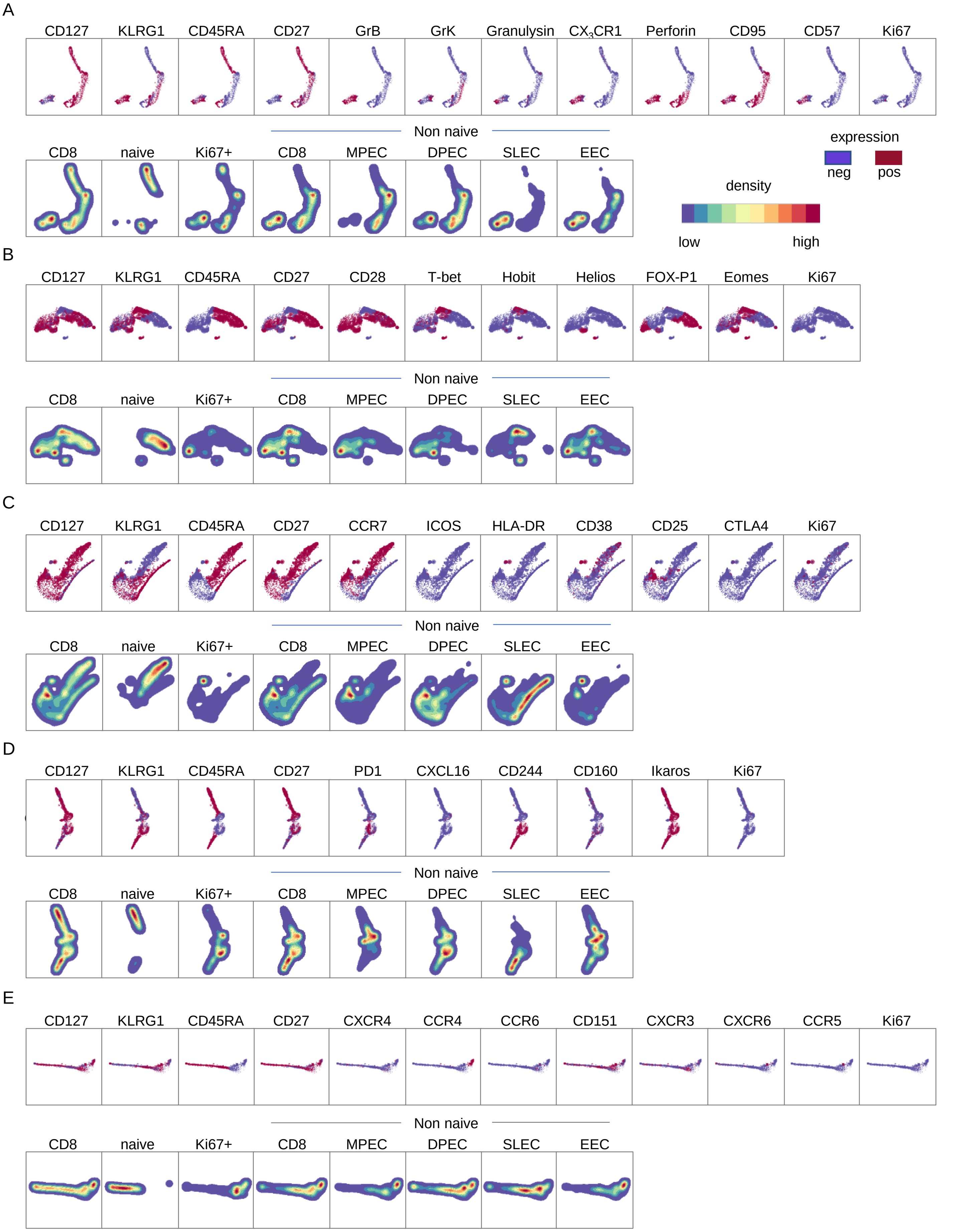

Figure S4. H-SNE analysis of non-naive KLRG1/IL-7Rα-defined $CD8^+CD3^+$ T cell subsets from figure 3 and S3 with Cytosplore.

Figure S4. H-SNE analysis of non-naive KLRG1/IL-7Rα-defined $CD8^+CD3^+$ T cell subsets from figure 3 and S4 with Cytosplore[1,2].

All compensated parameters of total live $CD8^+CD3^+$, naive $CD8^+CD3^+$, non-naive $CD8^+CD3^+$ cells and the non-naive KLRG1/CD127-defined subsets were exported from FlowJo (v10) and analysed in Cytosplore[1,2] (v4.5.0 - build 22.20.16.4836) with H-SNE.

Acitve parameters for H-SNE were

A) CD45RA, CD27, granzyme B, granzyme K, granulysin, CX3CR1, perforin, CD95, CD57 (table S1: panel 1)

B) CD45RA, CD27, CD28, T-bet, hobit, helios, FOX-P1, eomes (table S1: panel 2)

C) CD45RA, CD27, CCR7, ICOS, HLA-DR, CD38, CD25, CTLA-4 (table S1: panel 3)

D) CD45RA, CD27, PD1, CXCL16, CD244, CD160, ikaros (table S1: panel 4)

E) CD45RA, CD27, CXCR4, CCR4, CCR6, CD151, CXCR3, CXCR6, CCR5 (table S1: panel 5)

Ki-67, IL-7Rα and KLRG1 were analysed for their expression, but always excluded from the H-SNE analysis. All settings were left as automatically set by Cytosplore. Number of scales=5.

A-E) Upper row: expression of markers. Lower row: clusterdensity of total, naive, ki-67-expressing, non-naive $CD8^+CD3^+$ T cells and non-naive KLRG1/CD127-defined $CD8^+CD3^+$ T cell subsets (left to right).

Data shown (mean +-SD) are pooled from 1 independent experiments with n=10 donors per experiment.

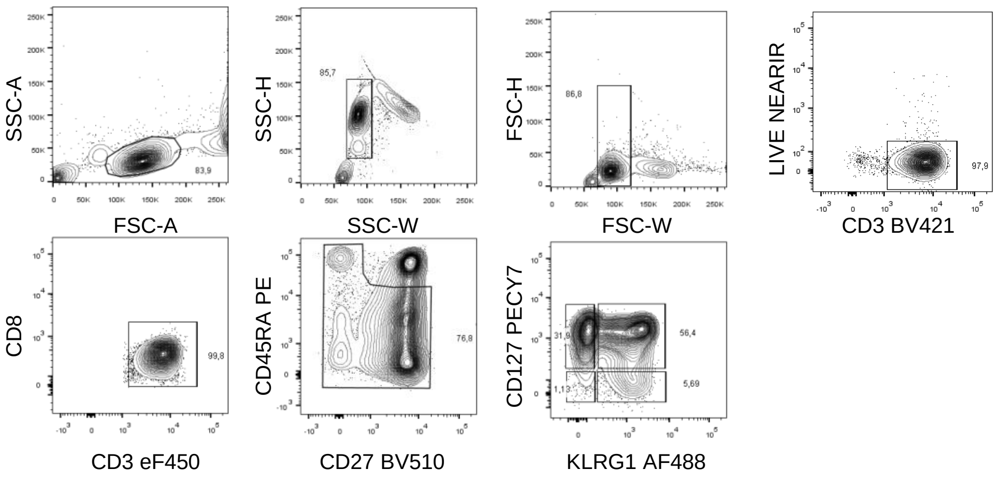

Figure S5. Sorting strategy of non-naive KLRG1/IL-7Rα-defined $CD8^+$ T cell subsets for RNAseq analysis. $CD8^+$ T cells were isolated from buffycoat derived PBMC with $CD8^+$ T cell beads (Miltenyi Biotec B.V.). Subsequently they were stained with a-CD3 eF450 (cloneSK7), a-CD127 (IL7Rα) PE-Cy7 (eBioRDR5) (eBioscience), a-CD8 APC (RPA-T8), a-CD45RA PE (HI100) (BD Biosciences), a-CD27 BV510 (O323) (Biolegend), a-KLRG1 AF488 (kind gift of HP Pircher) and live/dead fixable nearIR (Invitrogen) and sorted into all four KLRG1/IL-7Rα-defined $CD8^+CD3^+$ subsets on a FACS ARIA III sorter (BD).

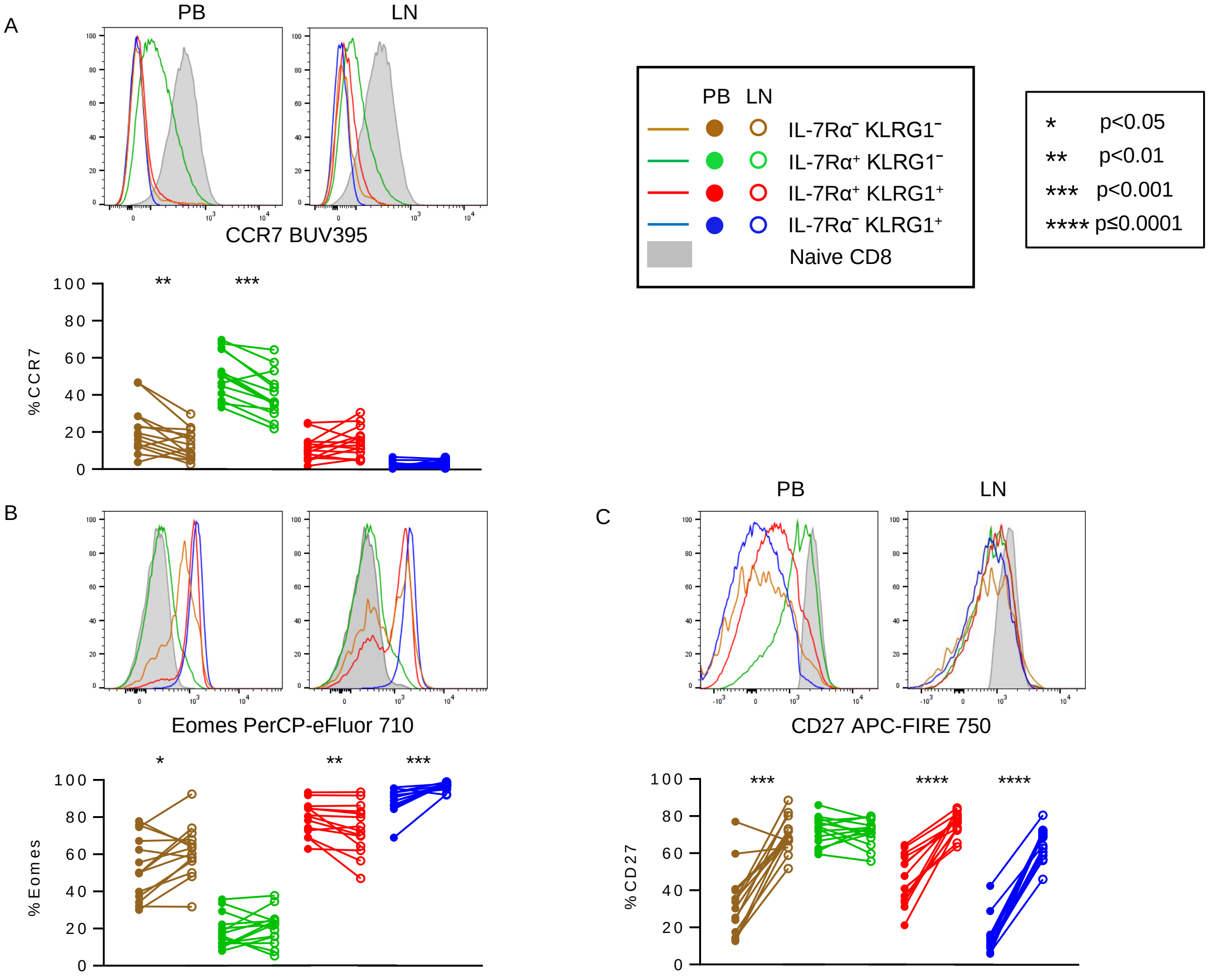

Figure S6: IL-7Rα^{hi}KLRG1^{lo} and non-cytotoxic memory traits are enriched in lymph nodes.
A-C) Representative examples (top) and percentages of expression of (A) CCR7, (B) eomes and (C) CD27 on KLRG1/IL-7Rα-defined subsets in non-naive CD8$^+$CD3$^+$ T cells of 14 PB with paired LN samples. Statistical analysis used: Wilcoxon matched-pairs signed rank test.
Data shown (mean +-SD) are pooled from/representative of 3 independent experiments with n=2, 7 and 5 donors per experiment.

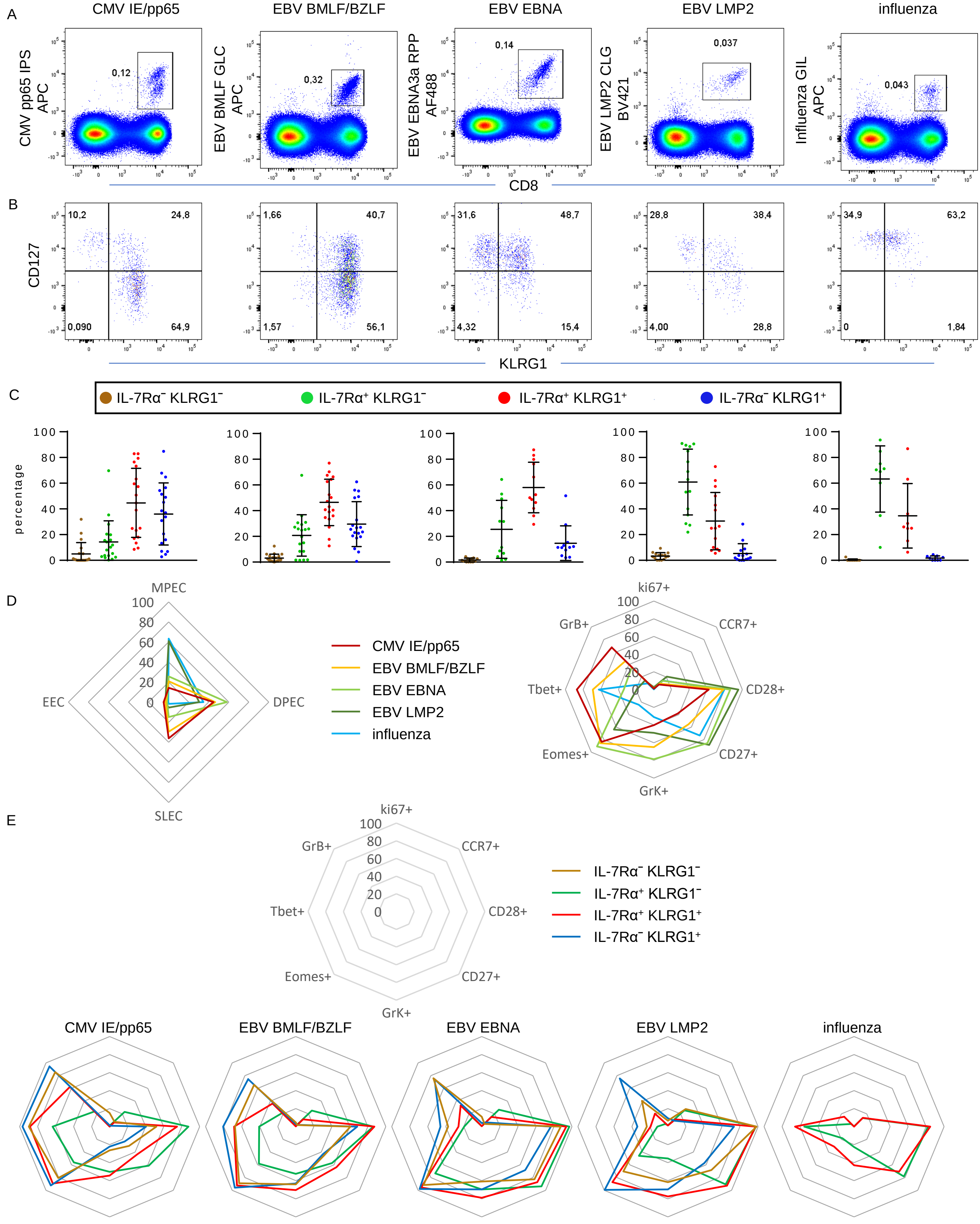

Figure s7: The IL-7Rα/KLRG1 division defines functionally distinct pools of virus specific CD8+ T cells: healthy individuals.

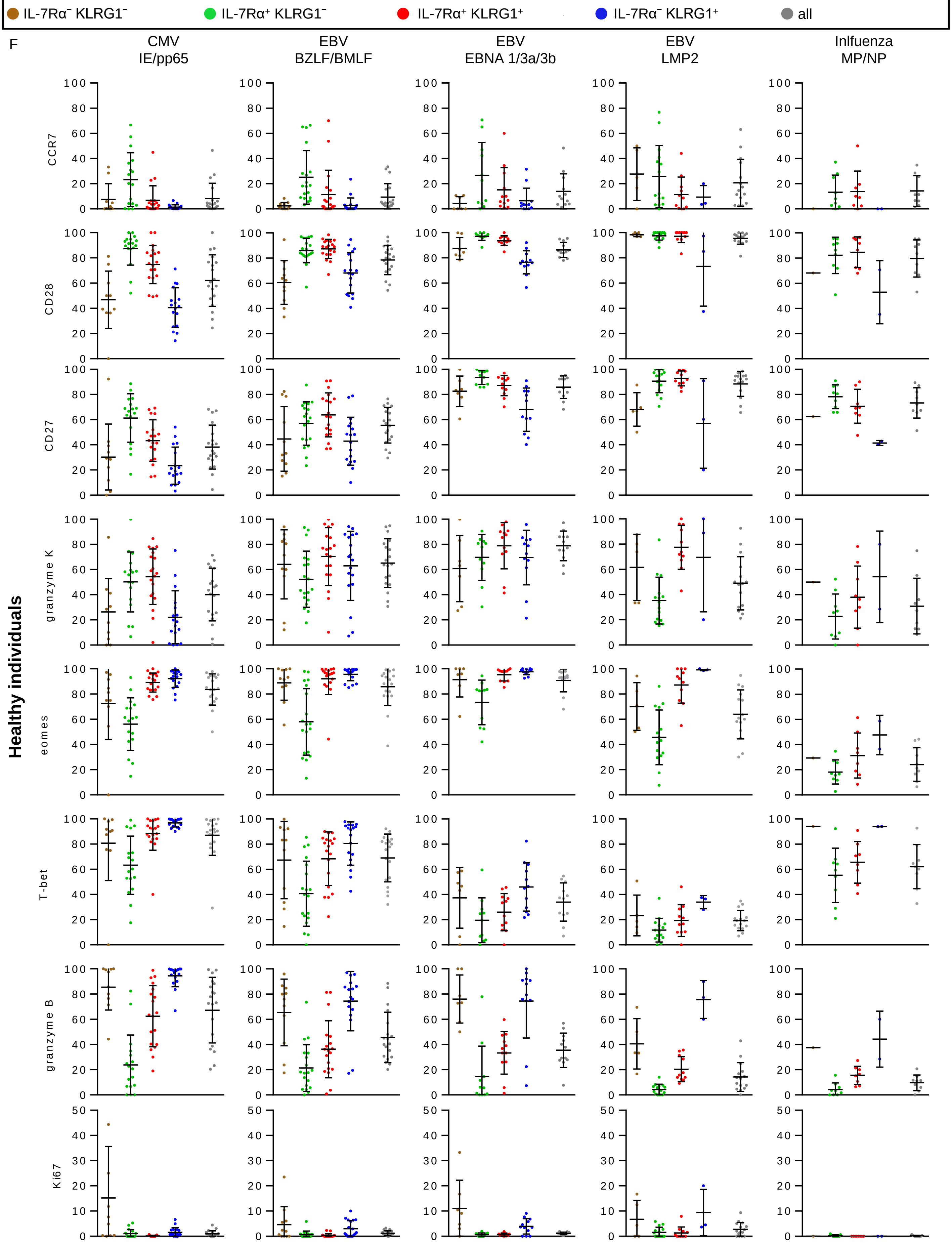

Figure s7 continued: The IL-7Rα/KLRG1 division defines functionally distinct pools of virus-specific CD8⁺ T cells: healthy individuals.

Figure s7: The IL-7Rα/KLRG1 division defines functionally distinct pools of virus specific CD8^{+} T cells: healthy individuals.

A-C) Representative examples of (a) CD8 and tetramer staining and (b) KLRG1 and IL-7Rα staining of CMV-IE/pp65-, EBV BMLF/BZLF-, EBV EBNA-, EBV-LMP2- and influenza-specific CD8^{+} T cells from one healthy donor and (c) distribution of KLRG1/IL-7Rα-defined subsets of the virus-specific CD8^{+} T cells (mean and SD)

D) Spiderplots of the distribution of KLRG1/IL-7Rα-defined subsets of the virus-specific CD8^{+} T cells (left) and the mean percentage of expression of ki67, CCR7, CD28, granzyme K, Tbet and granzymeB on total CMV-IE/pp65-, EBV BMLF/BZLF-, EBV-EBNA-, EBV LMP2 and influenza-specific CD8+ T cells (right; individual values can be found in S7f).

E) Spiderplot of the mean percentage of expression of ki67, CCR7, CD28, granzyme K, Tbet and granzyme B on KLRG1/IL-7Rα-defined subsets in CMV-IE/pp65-, EBV BMLF/BZLF-, EBV-EBNA-, EBV LMP2 and influenza-specific CD8+ T cells (individual values can be found in S7f).

F) Plots of individual values with mean and SD of CCR7, CD28, CD27, granzyme K, eomes, T-bet and granzyme B expression on CMV-IE/pp65-, EBV BMLF/BZLF-, EBV EBNA-, EBV-LMP2- and influenza-specific CD8+ T cells for each of the KLRG1/IL-7Rα-defined subsets.

(CMV-IE/pp65 (n=19), EBV BMLF/BZLF (n=19), EBV EBNA (n=12), EBV-LMP2 (n=15) and influenza (n=9)

Data shown (mean +-SD) are pooled from/representative of 3 independent experiments with n=8, 2 and 4 donors per experiment. 12 unique donors are shown.

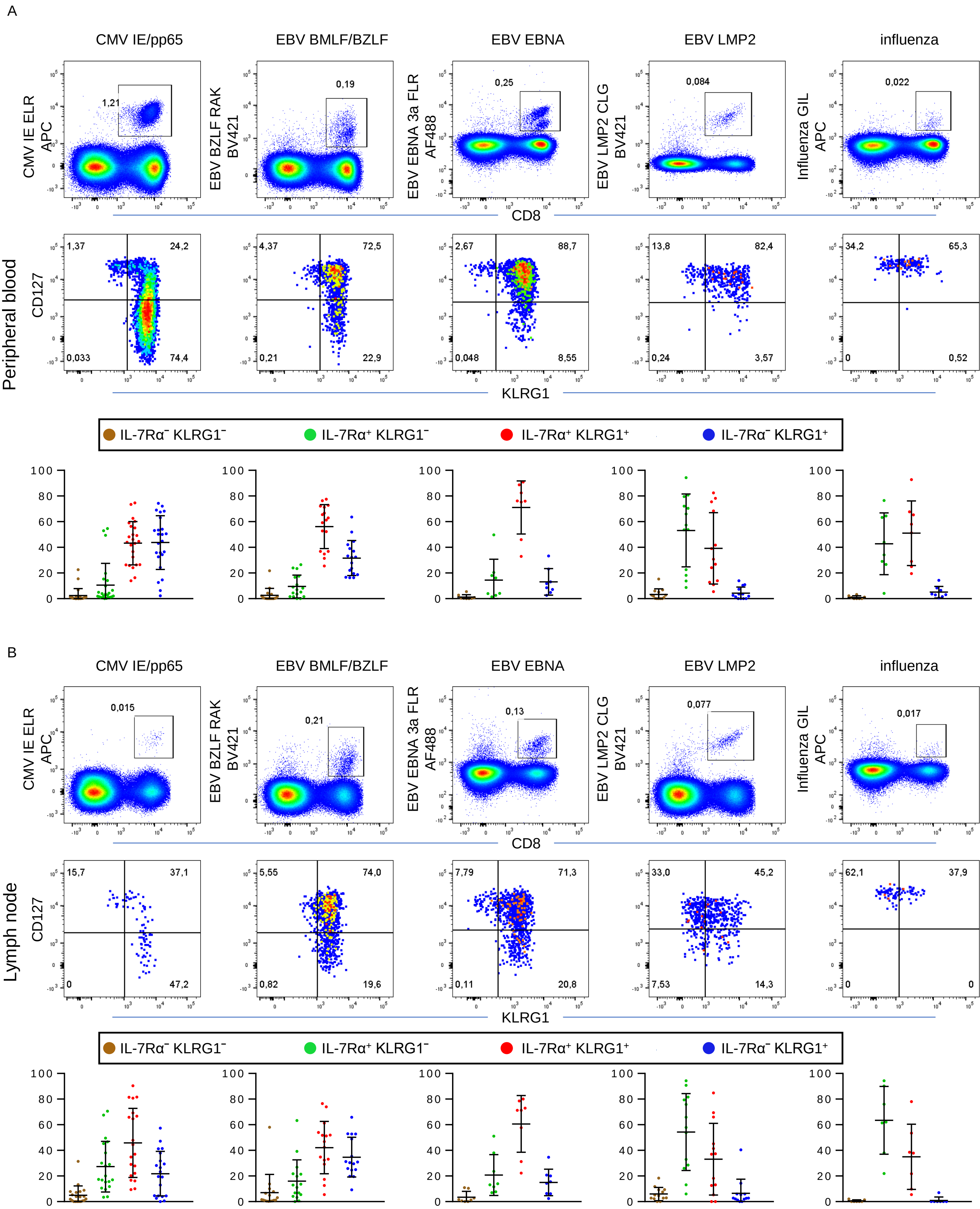

Figure s8a and b: The IL-7Rα/KLRG1 division defines functionally distinct pools of virus specific CD8+ T cells: peripheral blood vs lymph node.

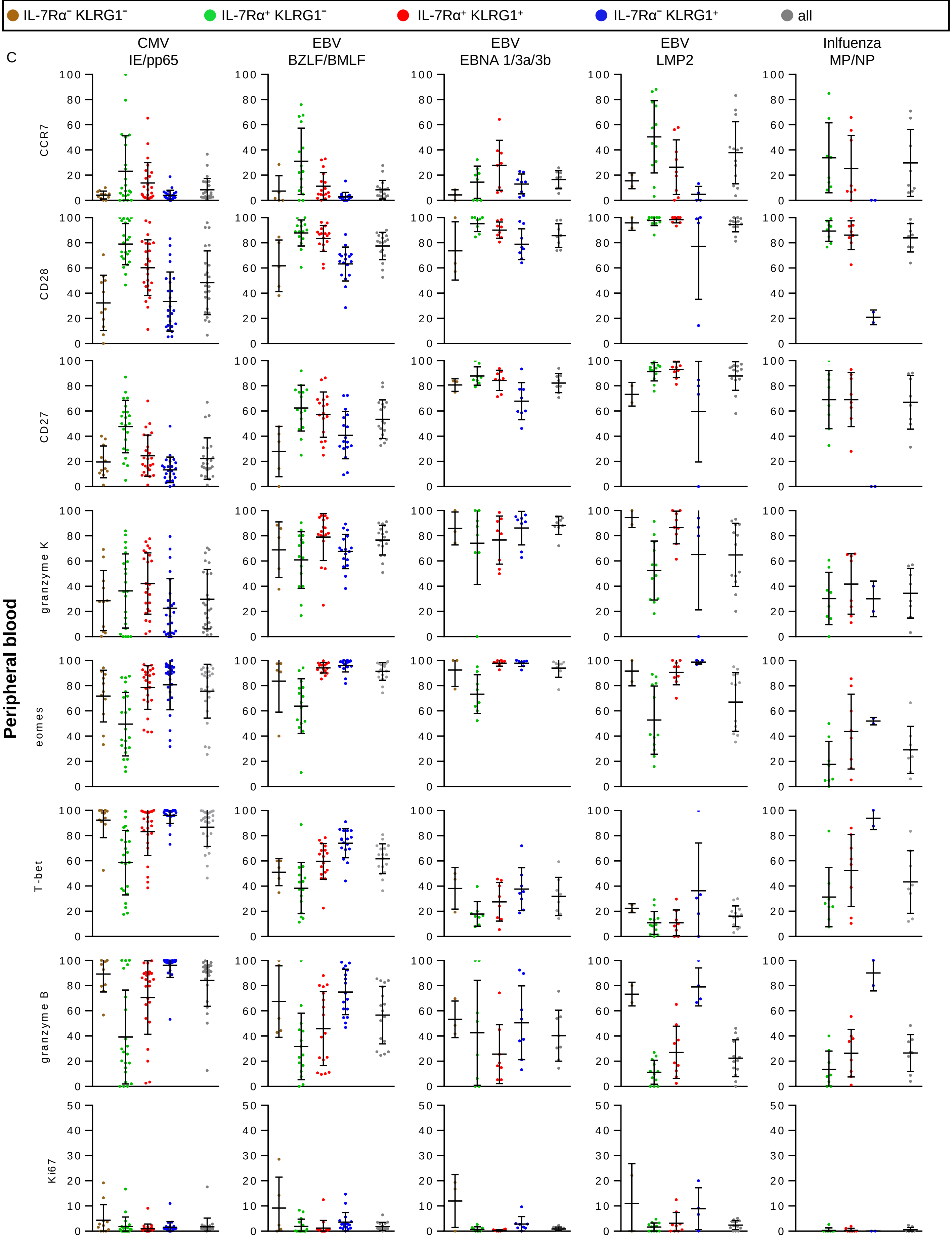

Figure s8c: The IL-7Rα/KLRG1 division defines functionally distinct pools of virus specific CD8+ T cells: peripheral blood vs lymph node.

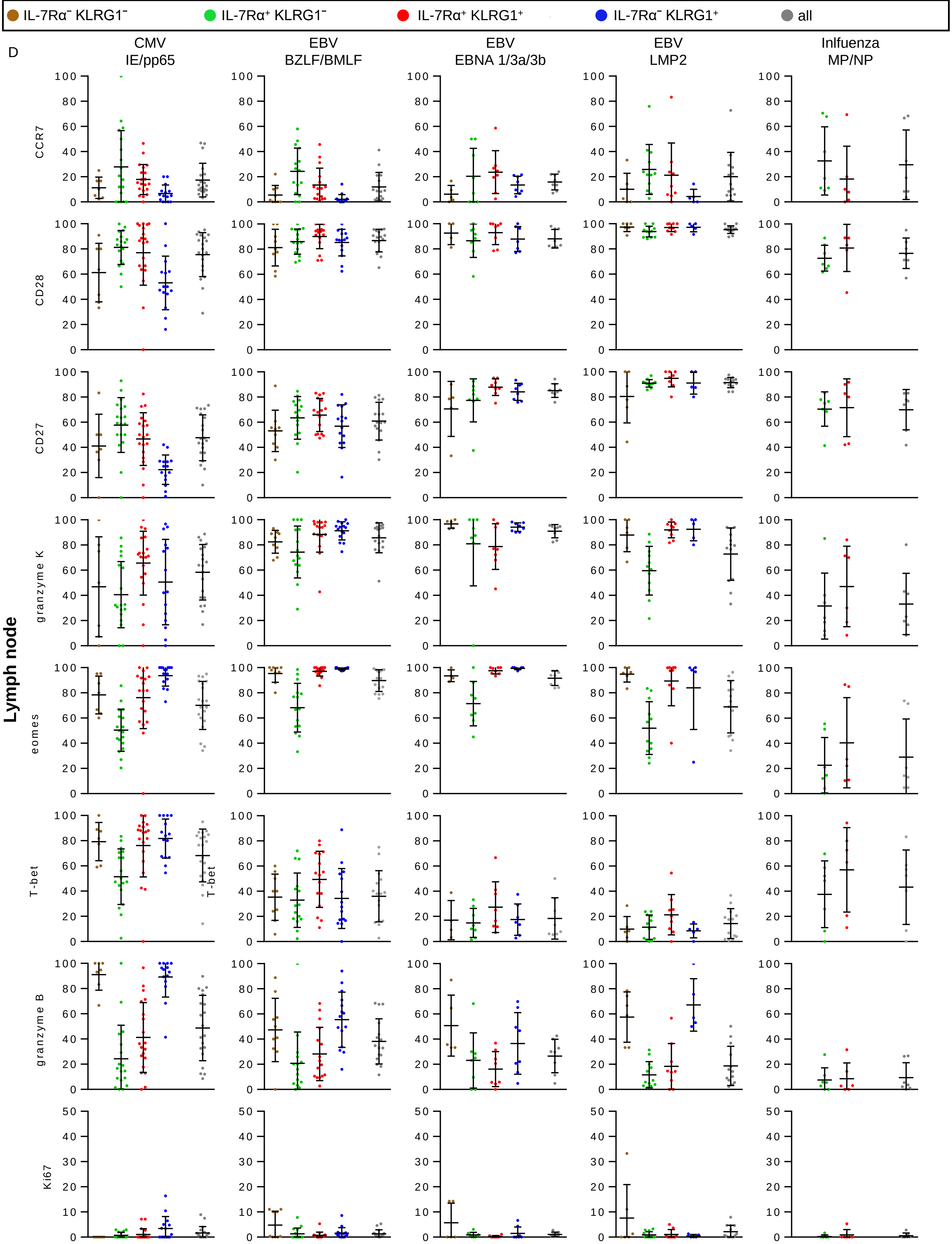

Figure s8d: The IL-7Rα/KLRG1 division defines functionally distinct pools of virus specific CD8+ T cells: peripheral blood vs lymph node.

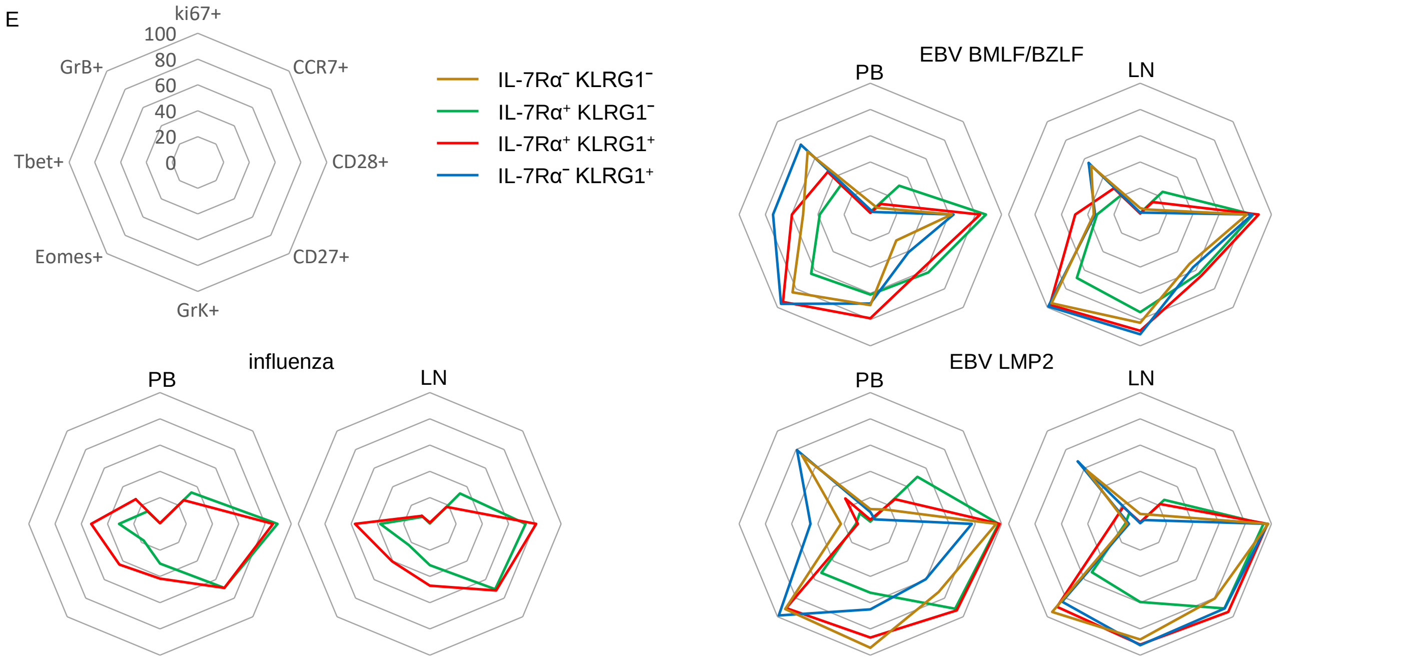

Figure s8E: The IL-7Rα/KLRG1 division defines functionally distinct pools of virus specific CD8+ T cells: peripheral blood vs lymph node.

Figure s8: The IL-7Rα/KLRG1 division defines functionally distinct pools of virus specific CD8$^+$ T cells.
A) Representative examples of CD8 vs tetramer staining (top) and KLRG1 vs IL-7Rα (second row) staining of peripheral blood (PB) derived CMV-IE/pp65-, EBV BMLF/BZLF-, EBV EBNA-, EBV-LMP2- and influenza-specific CD8$^+$ T cells from one individual and distribution of KLRG1/IL-7Rα-defined subsets of the virus-specific CD8$^+$ T cells (mean and SD, lower row).
B) Representative examples of CD8 vs tetramer staining(top) and KLRG1 vs IL-7Rα (second row) staining of paired lymph node (LN) derived CMV-IE/pp65-, EBV BMLF/BZLF-, EBV EBNA-, EBV-LMP2-and influenza-specific CD8$^+$ T cells) and distribution of KLRG1/IL-7Rα-defined subsets of the virus-specific CD8$^+$ T cells (mean and SD, lower row).
C) Plots of individual values with mean and SD of CCR7, CD28, CD27, granzyme K, eomes, T-bet, granzymeB, ki67 expressionon peripheral blood(PB) derived CMV-IE/pp65-, EBV BMLF/BZLF-, EBV EBNA-, EBV-LMP2-and influenza-specific CD8$^+$ T cells for each of the KLRG1/IL-7Rα-defined subsets.
D) Plots of individual values with mean and SD of CCR7, CD28, CD27, granzyme K, eomes, T-bet, granzymeB, ki67 expressionon lymphnode (LN) derived CMV-IE/pp65-, EBV BMLF/BZLF-, EBV EBNA-, EBV-LMP2-andinfluenza-specific CD8$^+$ T cells for each of theKLRG1/IL-7Rα-defined subsets.
E) Spiderplots of the percentages of expression of ki67 (c), CD28 (d), granzyme K (e), Tbet(f) and granzyme B (g) on KLRG1/IL-7Rα-defined subsets in EBV BMLF/BZLF-, EBV LMP2 and influenza-specific CD8$^+$ T cells (individual values can be found in S8c-d).
(CMV-IE/pp65 (n=24), EBV BMLF/BZLF (n=17), EBV EBNA (n=8), EBV-LMP2 (n=13) and influenza (n=8))
Data shown (mean +-SD) are pooled from/representative of 3 independent experiments with n=2, 7 and 5 donors per experiment.

Fig S7

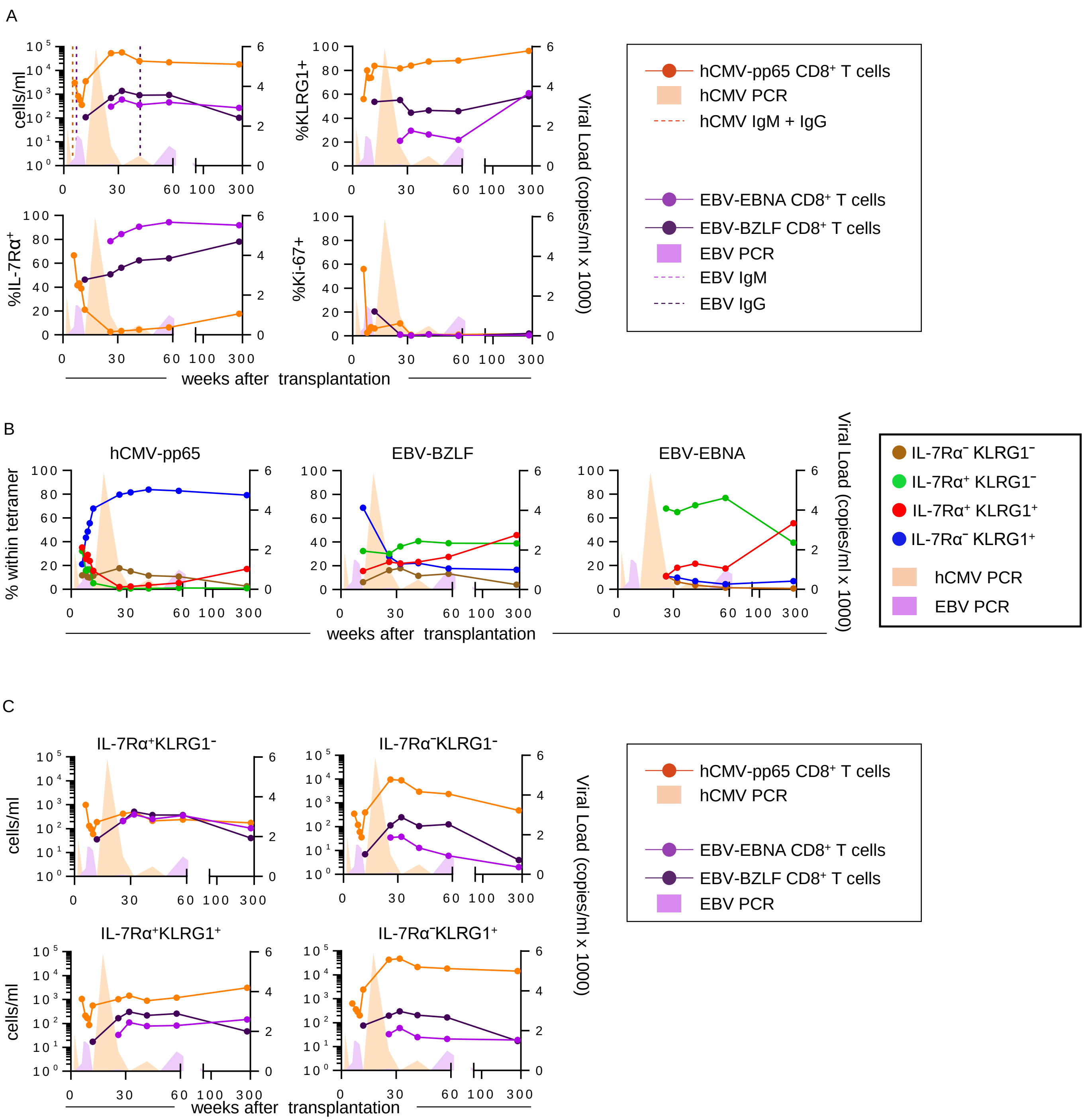

Fig S9. Dynamics and phenotype of hCMV- and EBV-specific $CD8^+$ T cells following primary hCMV and EBV in patient 2.
(A) Absolute numbers and percentage of KLRG1-expressing, IL-7Rα-expressing and Ki-67-expressing virus-specific $CD8^+$ T cells. (B) Percentages of KLRG1/IL-7Rα-defined subsets within the hCMV-, EBV-BZLF-and EBV-EBNA-specific $CD8^+$ T cells. (C) Absolute numbers of IL-7Rα$^+$KLRG1$^-$, IL-7Rα$^-$KLRG1$^-$, IL-7Rα$^+$KLRG1$^+$, IL-7Rα$^-$KLRG1$^+$ hCMV-, EBV-BZLF- and EBV-EBNA-specific $CD8^+$ T cells.
Data shown are 14 time points from 1 patient measured in 1 independent experiment.

Fig S8

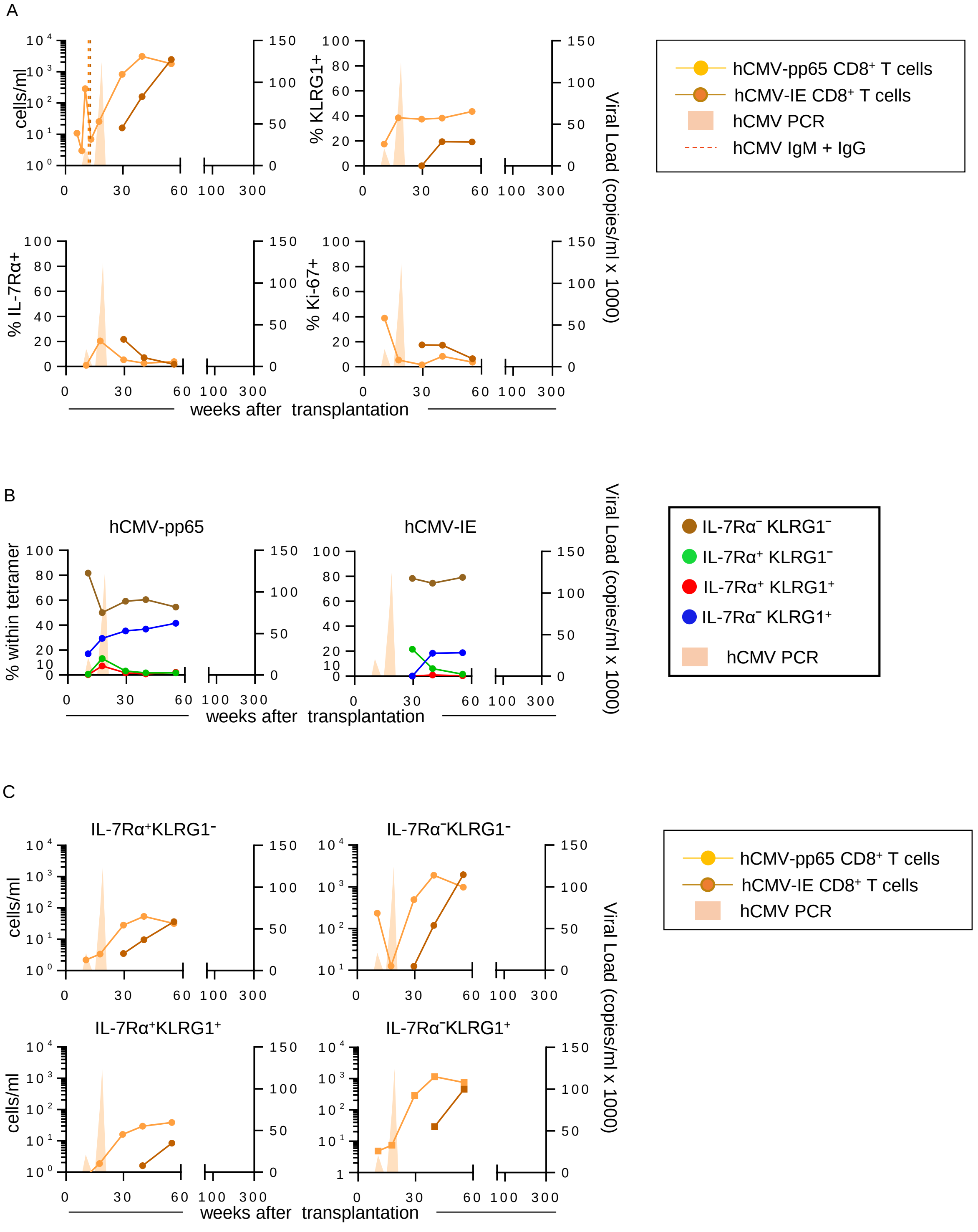

Figure S10. Dynamics and phenotype of hCMV-pp65- and IE-specific $CD8^+$ T cells following primary hCMV in patient 3.

(A) Absolute numbers and percentage of KLRG1-expressing, IL-7Rα-expressing and Ki-67-expressing virus-specific $CD8^+$ T cells. (B) percentages of KLRG1/IL-7Rα-defined subsets within the hCMV-, EBV-BZLF-and EBV-EBNA-specific $CD8^+$ T cells. (C) absolute numbers of IL-7Rα$^+$KLRG1$^-$, IL-7Rα$^-$KLRG1$^-$, IL-7Rα$^+$KLRG1$^+$, IL-7Rα$^-$KLRG1$^+$ hCMV-pp65- and hCMV-IE-specific $CD8^+$ T cells.

Data shown are 9 time points from 1 patient measured in 1 independent experiment.

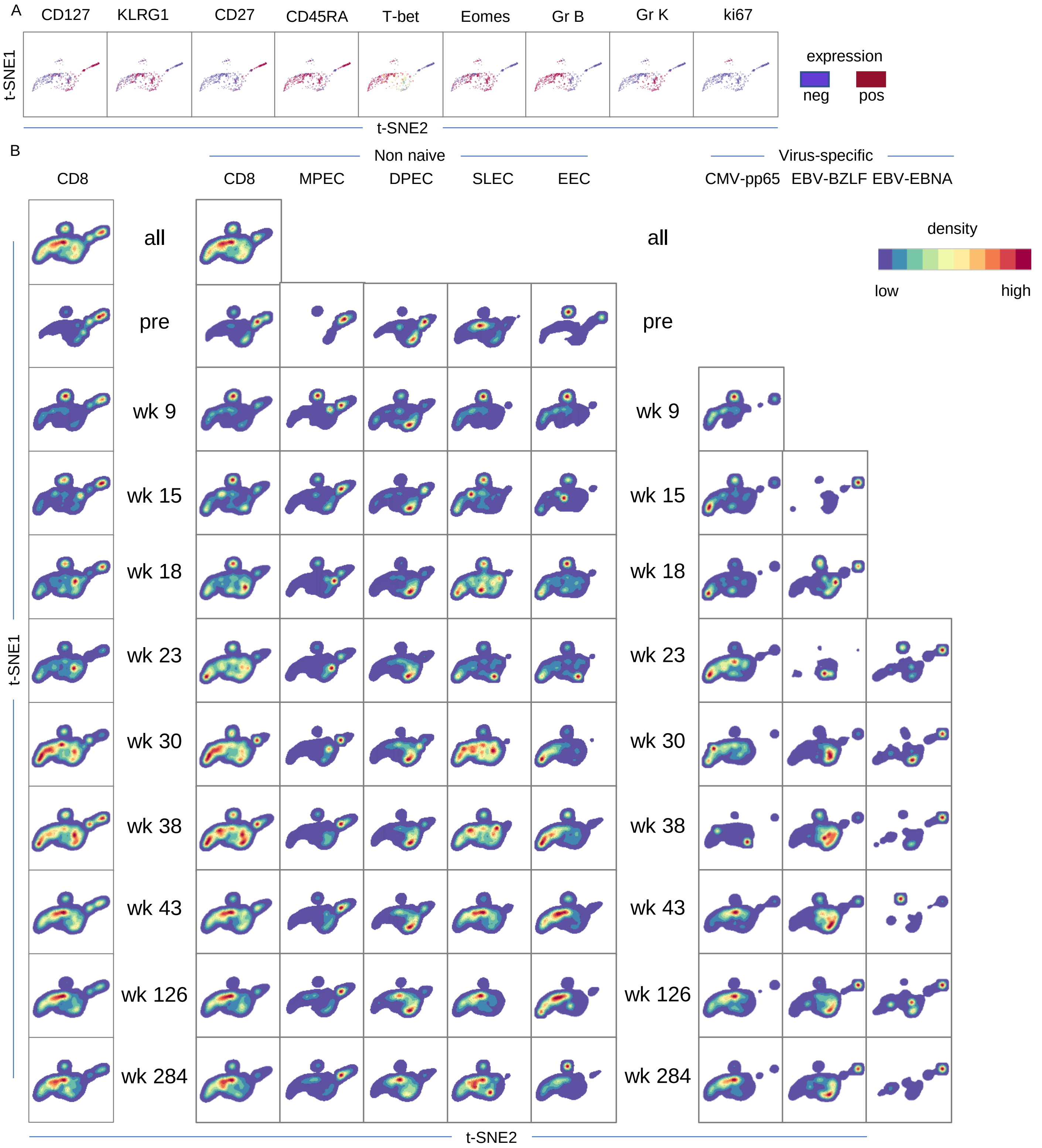

Figure S11. H-SNE analysis with, Cytosplore[1,2] of patient 1.

All compensated parameters of total live CD8⁺CD3⁺ cells, non-naive CD8⁺CD3⁺, non-naive KLRG1/CD127-defined subsets and virus-specific CD8⁺CD3⁺ cells were exported from FlowJo (v10) and analysed in Cytosplore (v4.5.0 - build 22.20.16.4836) with HSNE. Acitve parameters for HSNE were CD27, CD45RA, T-bet, Eomes, granzyme B, granzyme K and ki-67. Other parameters were only analysed for their expression but not used for H-SNE. All settings were left as automatically set by Cytosplore. Number of scales=5.

A: Expression of markers. B Clusterdensity of CD8, non-naive CD8, MPEC, DPEC, SLEC, EEC, CMV-pp65, EBV-BZLF1- and EBV-EBNA-specific CD8⁺ T cells (left to right) of all time points or specific timepoints before and after tranplantation (top to bottom).

Data shown are 10 time points from 1 patient measured in 1 independent experiment.

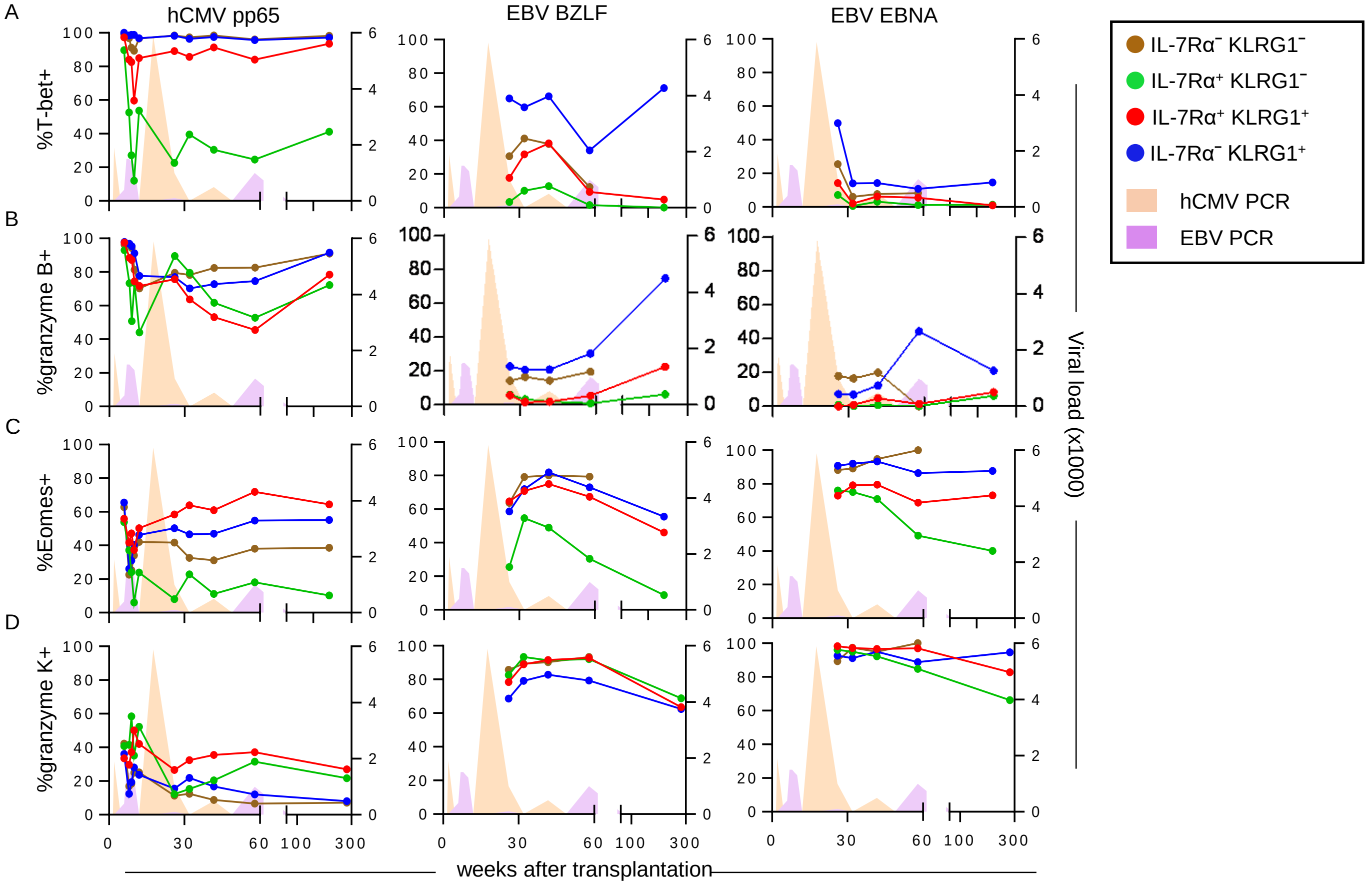

Figure S12. Phenotype of the KLRG1/IL-7Rα defined subsets within hCMV- and EBV-specific CD8$^+$ T cells following primary hCMV and EBV in patient 2.

(A-D) Percentages of (A) T-bet-expressing, (B) granzyme B-expressing, (C) Eomes-expressing and (D) granzyme K-expressing cells within the KLRG1/IL-7Rα defined subsets in hCMV-, EBV-BZLF- and EBV-EBNA-specific CD8$^+$ T cells.

Data shown are 14 time points from 1 patient measured in 1 independent experiment.

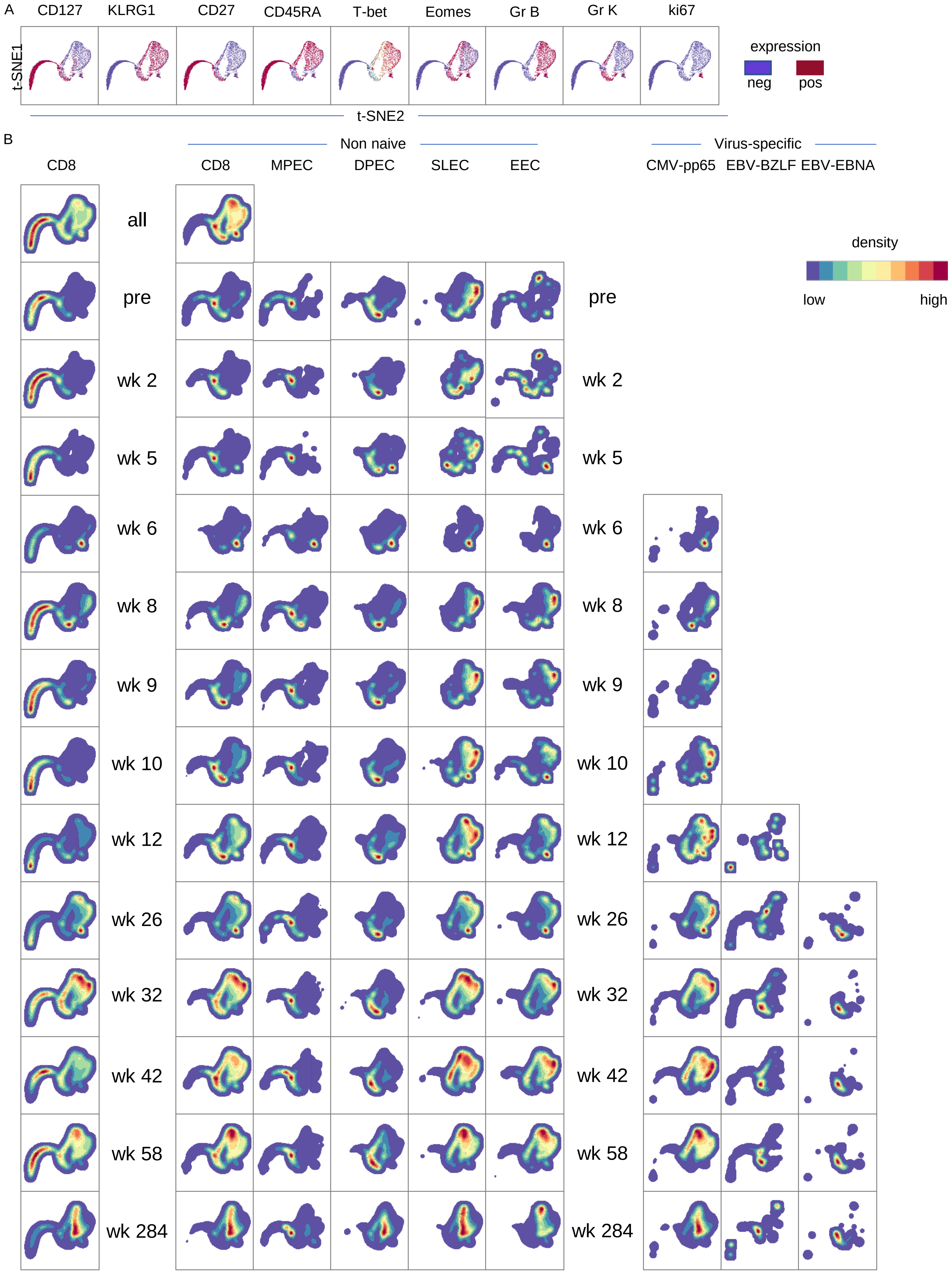

Figure S13. H-SNE analysis with, Cytosplore[1,2] of patient 2.

Figure S13. H-SNE analysis with, Cytosplore[1,2] of patient 2.

All compensated parameters of total live $CD8^+CD3^+$ cells, non-naive $CD8^+CD3^+$, non-naive KLRG1/CD127-defined subsets and virus-specific $CD8^+CD3^+$ cells were exported from FlowJo (v10) and analysed in Cytosplore (v4.5.0 - build 22.20.16.4836) with HSNE. Acitve parameters for HSNE were CD27, CD45RA, T-bet, Eomes, granzyme B, granzyme K and ki-67. Other parameters were only analysed for their expression but not used for H-SNE. All settings were left as automatically set by Cytosplore. Number of scales=5.

A: Expression of markers. B Clusterdensity of CD8, non-naive CD8, MPEC, DPEC, SLEC, EEC, CMV-pp65, EBV-BZLF1- and EBV-EBNA-specific $CD8^+$ T cells (left to right) of all time points or specific timepoints before and after tranplantation (top to bottom).

Data shown are 14 time points from 1 patient measured in 1 independent experiment.

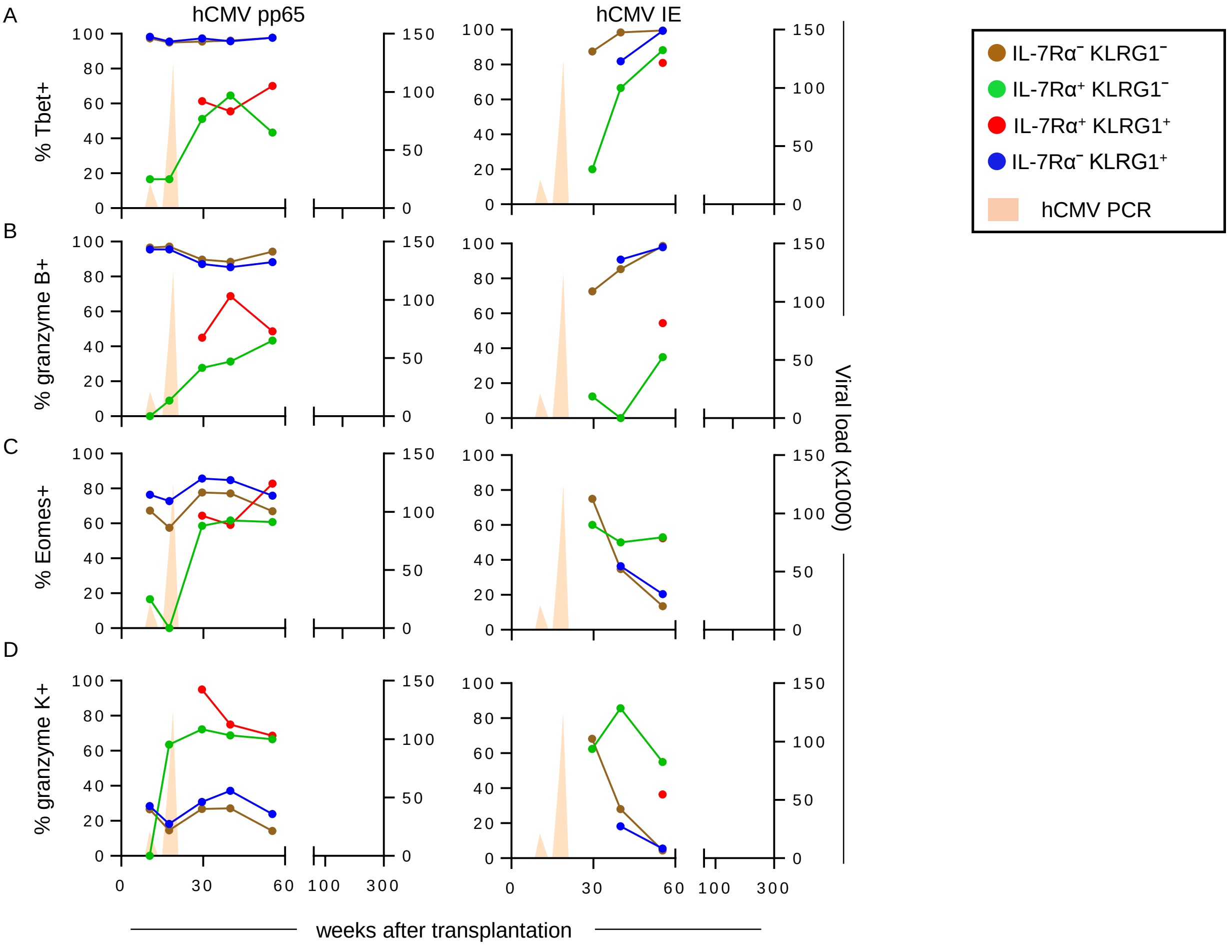

Figure S14: Phenotype of the KLRG1/IL-7Rα defined subsets within hCMV-specific CD8⁺ T cells following primary hCMV in patient 3.
(A-D) Percentages of (A) T-bet-expressing, (B) granzyme B-expressing, (C) Eomes-expressing and (D) granzyme K-expressing cells within the KLRG1/IL-7Rα defined subsets in hCMV-pp65 and hCMV-IE-specific $CD8^+$ T cells. Data shown are 9 time points from 1 patient measured in 1 independent experiment.

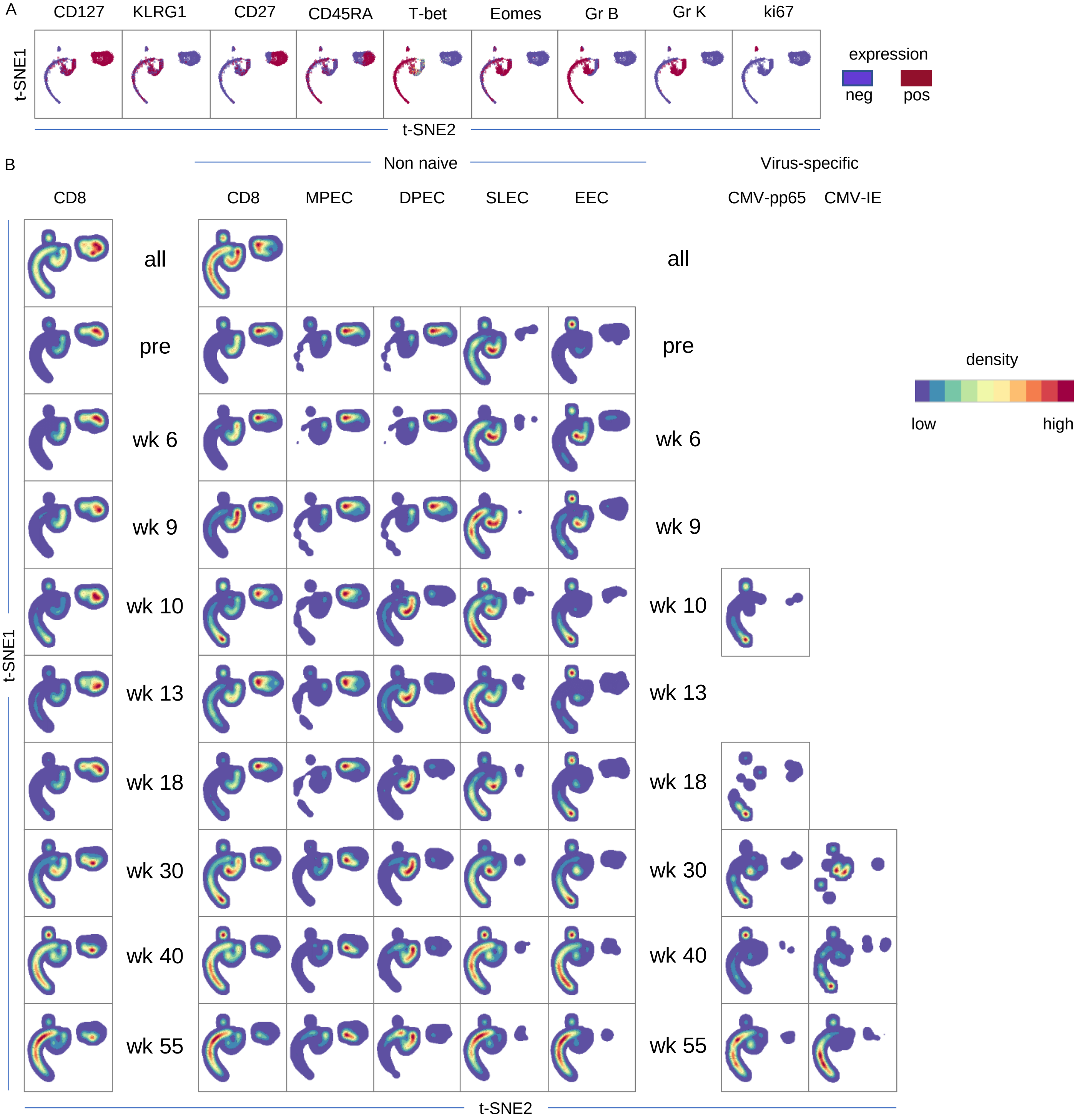

Figure S15. H-SNE analysis with, Cytosplore[1,2] of patient 2.

All compensated parameters of total live CD8⁺CD3⁺ cells, non-naive CD8⁺CD3⁺, non-naive KLRG1/CD127-defined subsets and virus-specific CD8⁺CD3⁺ cells were exported from FlowJo (v10) and analysed in Cytosplore (v4.5.0 - build 22.20.16.4836) with HSNE. Acitve parameters for HSNE were CD27, CD45RA, T-bet, Eomes, granzyme B, granzyme K and ki-67. Other parameters were only analysed for their expression but not used for H-SNE. All settings were left as automatically set by Cytosplore. Number of scales=5.

A: Expression of markers. B Clusterdensity of CD8, non-naive CD8, MPEC, DPEC, SLEC, EEC, CMV-pp65 and CMV-IE-specific CD8⁺ T cells (left to right) of all time points or specific timepoints before and after tranplantation (top to bottom).

Data shown are PBMC from 9 time points from 1 patient measured in 1 independent experiment.

1. T. Höllt, N. Pezzotti, V. van Unen, F. Koning, E. Eisemann, B. Lelieveldt, and A. Vilanova. Cytosplore: Interactive Immune Cell Phenotyping for Large Single-Cell Datasets. *Computer Graphics Forum (Proceedings of EuroVis),* 35(3): pp. 171—180, 2016.
2. V. van Unen, T, Höllt, N. Pezzotti, N. Li, M. Reinders, E. Eisemann, A. Vilanova, F. Koning, and B. Lelieveldt. Visual Analysis of Mass Cytometry Data by Hierarchical Stochastic Neighbor Embedding Reveals Rare Cell Types. *Nature Communications,* 2017
